# Supplementary material for: Powerful detection of polygenic selection and evidence of environmental adaptation in US beef cattle
Source: PLoS Genet. 2021 Jul 22;17(7):e1009652. doi: 10.1371/journal.pgen.1009652 (PMC8297814; doi:10.1371/journal.pgen.1009652)
Supplement: S1 Text — This file includes supplementary text, Tables A-J, and SI References. (DOCX) [file pgen.1009652.s001.docx]

S1 Text: Supplementary Information for

**Powerful detection of polygenic selection and environmental adaptation in US beef cattle**

Troy N. Rowan, Harly J Durbin, Christopher M. Seabury, Robert D. Schnabel, Jared E. Decker*

* deckerje@missouri.edu

**This PDF file includes:**

Supplementary text

Tables A to J

SI References

**Supplementary Text**

GPSM detects signatures of polygenic selection across and within three populations of U.S. Beef Cattle

In addition to the locus undergoing a massive allele frequency shift on BTA28 (lead SNP *rs1762920*), we identify 6 other loci under selection in all three populations. (**Table E**). These shared GPSM signals suggest that not only are there similar selection pressures, but common genomic architectures under selection in these three populations. While we identify significantly more population-specific GPSM signatures, shared signatures are of interest as they are likely serving an important role in all breeds of beef cattle. We discuss these loci and their potential functions in beef cattle that are driving allele frequency changes.

The largest genomic region detected by GPSM lies at the end of BTA1 (157.5 Mb - 158.5 Mb). The lead SNP in this peak (*rs1755753*) lies within the long non-coding RNA (lncRNA) *LOC112448253*. This lncRNA has not been previously associated with any traits or function in cattle. This region contains dozens of other potential candidate genes. This selected region is also immediately upstream of *PRDM9*, a modulator of recombination in most mammalian species, including cattle [[1–3]](https://paperpile.com/c/AKVIGO/obSIr+XPXM6+dEvkm). While no variants within PRDM9 reach genome-wide significance, variants ~10.7 kb from the TSS are responding to selection. Selection on *PRDM9* could increase average recombination rate or allow novel motif binding in order to create novel favorable haplotype combinations [[4]](https://paperpile.com/c/AKVIGO/BRGnr).

We identified six other common genomic regions under strong selection in all three populations, encompassing 106 statistically significant markers. Another 90 SNPs overlap in at least two populations, corresponding to 15 additional genomic regions under selection (**Table E**). All three populations have been selected for increased growth traits over the last 50 years [[5]](https://paperpile.com/c/AKVIGO/zicE8), and GPSM identifies two genomic regions that have been previously associated with feed efficiency and growth traits. The common peak on BTA12 (lead SNP *rs1389713*) is ~182 kb upstream of the *DACH1* (Dachshund homolog 1), a transcription factor associated with post-weaning gain, various indicators of feed efficiency [[6,7]](https://paperpile.com/c/AKVIGO/zHr9Z+uSWrT), and backfat thickness [[8]](https://paperpile.com/c/AKVIGO/d67MT) in cattle. The shared GPSM peak on BTA14 resides near a known QTL for post-weaning gain near the gene *LRP12* (LDL receptor related protein 12) [[9]](https://paperpile.com/c/AKVIGO/zrOFa).

In addition to selection on loci that appear directly involved in growth and efficiency, we identify multiple selection targets likely involved in aspects of immune function. A shared significant peak on BTA2 (lead SNP *rs1080110*) resides within the *ARHGAP15* gene (Rho GTPase Activating Protein 15) that is essential for Trypanosomiasis resistance in African cattle populations [[10–12]](https://paperpile.com/c/AKVIGO/InarH+hOSw2+tItrU). Though Trypanosomiasis and other tsetse fly-transmitted diseases are restricted to Africa, genetic tolerance to similar immune disturbances may account for the positive selection observed in these three American cattle populations. Variants within *ADORA1* (Adenosine A1 Receptor) are also detected as being under selection by GPSM. In cattle, *ADORA1* plays a role in the activation of polymorphonuclear neutrophilic leukocytes, which are important for peripartial immune responses in cattle [[13]](https://paperpile.com/c/AKVIGO/dTsC), and likely play roles in other immune functions. *ADORA1* and other purinergic receptors play an important role in bone metabolism [[14]](https://paperpile.com/c/AKVIGO/y8Bfi) and likely growth in cattle. This signature within *ADORA1* also spans a potentially regulatory region for *MYBPH*, an important gene in muscle formation and development, and another potential target of selection [[15]](https://paperpile.com/c/AKVIGO/Hemd). In this case and others, we identify multiple logical candidate genes within regions undergoing selection. We report a complete listing of significant SNPs and candidate genes from GPSM analyses of each population in (**S2 Table**).

We observe 22 genomic regions that are changing in frequency in at least two of our datasets. In addition to these shared loci, we identify evidence of shared networks and genetic architectures under selection (**Table E**). To discern common biological pathways and processes under selection across multiple populations, we identified genes within 10 kb of SNPs identified by GPSM in at least two populations and performed a gene enrichment analysis in ClueGO. Using the 46 genes residing in or near the 29 shared GPSM signatures identified by at least two datasets identified multiple biological processes undergoing selection. The most significant pathways involved G-protein coupled signaling (Benjamini–Hochberg adjusted p-value = 0.038) and purinergic receptor signaling pathways (Benjamini–Hochberg adjusted p-value = 0.034, associated genes *ADORA1* and *P2RY8*). Purinergic receptors have been identified as important drivers of immune responses in cattle [[13]](https://paperpile.com/c/AKVIGO/dTsC). Selection on immune pathways is likely driven by the increased production efficiency of healthy calves [[16,17]](https://paperpile.com/c/AKVIGO/Bv6Ay+xXhXu). Shared selection on *SLC2A5* and *BIRC5* point towards biological pathways involved in sensing carbohydrate, hexose, and monosaccharide stimuli (Benjamini-Hochberg adjusted p-value = 0.01). We also expect that an enhanced metabolic response to carbohydrates would result in increased animal efficiency. Finally, genes involved in the regulation of arterial blood pressure (Benjamini–Hochberg adjusted p-value = 0.045: *ADORA1*, *SLC2A5*) made up the lone other significant gene class under selection across all three populations. Gene enrichment analysis within populations also identified population-specific pathways and processes under selection. In Simmental cattle we detect 23 GPSM candidate genes involved in olfactory transduction. While not related directly to growth traits, olfactory receptors have been identified as selection targets in many mammalian species, including cattle [[18–20]](https://paperpile.com/c/AKVIGO/4V5fB+3uWbY+Db06R). This rapidly-evolving class of genes is also associated with growth and carcass traits, suggesting a wide range of functions that are not limited to detecting smell [[9,21]](https://paperpile.com/c/AKVIGO/zrOFa+UeBVu).

envGWAS identifies adaptive pathways and processes.

Local adaptation is likely highly complex and controlled by many areas of the genome. Though we detected minimal overlap in candidate genes across datasets, we identified multiple conserved biological processes and pathways that appear to play roles in local adaptation across populations.

Though there was minimal candidate gene overlap between continuous and discrete envGWAS (19 of 187 total envGWAS candidate genes in Red Angus), we identified many shared pathways and gene ontologies. In most cases where we observed GO or pathway overlap, statistical significance was greater for the discrete envGWAS analysis, simply due to the difference in number of provided genes (168 vs 38). Most of the shared terms and pathways were driven by the genes *BAD*, *EFNA5*, *LRRC4C*, *MARK2*, *PLCB3*, and *PRKG2* detected in both analyses. In these overlapping terms, additional genes from the discrete zone envGWAS further supplemented the identified terms.

We performed gene enrichment analyses with gene lists from all univariate and multivariate, discrete and continuous envGWAS analysis in each population. This allowed sufficiently large gene lists to identify potentially adaptive pathways and processes. We were particularly interested in identifying shared pathways and processes between populations since the number of shared genomic regions was low. Across all three populations, we consistently identified the “axon guidance” pathway, and numerous GO terms relating to axon development and guidance under region-specific selection. Ai et al. (2015) [[22]](https://paperpile.com/c/AKVIGO/XrRwZ) suggested that axon development and migration in the central nervous system is essential for the maintenance of homeostatic temperatures by modulating heat loss or production [[23]](https://paperpile.com/c/AKVIGO/aUEvi). The direction and organization of axons is an essential component of the olfactory system which is frequently implicated in environmental adaptation through the recognition of local environmental cues [[24,25]](https://paperpile.com/c/AKVIGO/xEqbM+Yxs7). Other pathways identified across all three datasets include “cholinergic synapse”, “glutamatergic synapse”, and “platelet activation”. Cholinergic signaling drives cutaneous vascular responses to heat stress in humans [[26–28]](https://paperpile.com/c/AKVIGO/3IdfL+MH9ju+8S1Hj). Additionally, cholinergic receptors act as the major neural driver of sweating in humans [[29]](https://paperpile.com/c/AKVIGO/4IZKu). Glutamatergic synapses are involved in neural vasoconstriction [[30,31]](https://paperpile.com/c/AKVIGO/ddKIO+9nYyW). “Retrograde endocannabinoid signaling”, “dopaminergic synapse”, “GABAergic synapse”, and “serotonergic synapse” pathways are also significantly enriched by envGWAS candidate genes. Nearly all of these important neural signaling pathways are also enriched in a series of gene-by-environment interaction GWAS for birth weight, weaning weight, and yearling weight in Simmental cattle by [[32]](https://paperpile.com/c/AKVIGO/z7Wql). Taken together, these results suggest that pathways involved in neuron development and neurotransmission are essential components of local adaptation in cattle. Temperature homeostasis is largely controlled by the central nervous system [[33]](https://paperpile.com/c/AKVIGO/mHGXG), making environment-specific selection on these pathways an efficient way for populations to adapt.

Other pathways identified by envGWAS appear to play important roles in vasodilation and vasoconstriction. Relaxin signaling was identified in both Red Angus and Simmental populations as a locally adaptive pathway under selection. Relaxin, initially identified as a pregnancy-related hormone, is an important modulator of vasodilation [[34]](https://paperpile.com/c/AKVIGO/ytYW3). In Simmental this pathway association originates from local adaptation signatures near the genes *COL1A1*, *MAPK10*, and *PRKACA* identified both in our discrete multivariate envGWAS and in the Desert ecoregion univariate analysis. Relaxin signaling was also identified in Red Angus, but with four entirely different genes (*LOC529425*, *PLCB3*, *PRKACB*, *VEGFB*). While all four of the Red Angus genes were identified in multivariate analyses, we identify two of them in the Desert ecoregion univariate envGWAS. Vasodilation is an essential component of physiological temperature adaptation in cattle and other species [[35–37]](https://paperpile.com/c/AKVIGO/rJYEt+kQDQC+Lu4mA). The ability to mount a physiological response to heat stress has a direct impact on cattle performance. Heat stressed cattle have decreased feed intake, slower growth rates, and decreased fertility [[38]](https://paperpile.com/c/AKVIGO/CFRWj). The Renin secretion pathway, which is also directly involved in vasoconstriction was identified in Red Angus (*ADRB1*, *PLCB3*, *PRKACB*, *PRKG2*), and has also been previously implicated in physiological responses to heat stress in cattle [[39]](https://paperpile.com/c/AKVIGO/nTNfj). In each population we identify multiple biological processes related to the regulation of insulin secretion. Insulin secretion is elevated in heat stressed cattle [[40]](https://paperpile.com/c/AKVIGO/1wF6W) and pigs [[41]](https://paperpile.com/c/AKVIGO/tZSd5), suggesting that it plays a role in metabolism and thermoregulation. Insulin secretion in response to the presence of glucose may also be related to different diets and forage availability along these continuous environmental gradients [[42]](https://paperpile.com/c/AKVIGO/l6JCI).

Other pathways identified by envGWAS candidate genes from Simmental point towards the immune system’s role in local adaptation. “Th1 and Th2 cell differentiation” and “Th17 cell differentiation” were significant in a KEGG pathway analysis. The development of these cell types is essential for adaptive immune responses [[43,44]](https://paperpile.com/c/AKVIGO/JhZ8Q+WxSKH). These signals were driven in part by region-specific allele frequency differences in or near *MAPK10* an innate immunity gene identified in human studies as an adaptive target of selection [[45]](https://paperpile.com/c/AKVIGO/Wcnik). We also identify multiple cardiac-related pathways from Simmental envGWAS genes (“Dilated cardiomyopathy”, “Arrhythmogenic right ventricular cardiomyopathy”, “Hypertrophic cardiomyopathy”) driven by a pair of related genes *SGCA* and *SGCD*. Like other circulatory-related pathways, the alleles driving this signal were identified in both multivariate and Desert ecoregion envGWAS. We expect that these cardiac-related pathways, like renin secretion and relaxin signaling affect the efficiency of circulation and improved temperature homeostasis when exposed to heat or cold stress. Selection on cardiovascular function is also likely a central component of adaptation to high altitude [[46]](https://paperpile.com/c/AKVIGO/08tFh).

Tissue Set Enrichment Analysis

To further disentangle the biological basis of GPSM and envGWAS adaptive signatures, we performed a series of Tissue Set Enrichment Analyses (TSEA) based on gene expression data from humans and worms (*C. elegans*). These analyses identified tissues in which our envGWAS candidate genes were preferentially expressed. Our candidate gene lists for each population consisted of annotated cattle genes within 10 kb of a significant GPSM SNPs or SNPs identified in any of our multivariate or univariate envGWAS analysis. In using expression data from other species, non-orthologous cattle genes are not included in enrichment analyses.

Using GPSM candidate genes in TSEA identified enriched tissues that correspond to population-specific production traits known to be under selection. Using gene expression measures from the GTEx pilot dataset [[47]](https://paperpile.com/c/AKVIGO/vc9jV) in the pSI R package [[48]](https://paperpile.com/c/AKVIGO/j9rRl) we identify suggestive enriched expression in various reproductive-related tissues from the Red Angus GPSM candidate gene set. We observe suggestive enrichments (p < 0.1) for human breast, ovary, pituitary, and uterus tissues (**S4 and S5 Tables**) among others. An analysis of this gene list with gene expression data from the Human Protein Atlas also identified significantly enriched expression in cervix, uterus, and brain tissues (FDR-corrected p-value < 0.1) and suggestive expression fold change in the ovary. These results provide further evidence that selection on fertility and reproductive traits have been ongoing in the Red Angus population over the last ~10 generations (Red Angus Association of America EPD trends <https://redangus.org/genetics/epd-trends/>). Further, using Gelbvieh GPSM candidate gene sets, we identify enriched expression in numerous tissues including skeletal muscle, nerve, thyroid and adipose tissue. These enriched tissues align with known ongoing selection for increased growth and carcass quality. Despite the numerous enriched pathways and processes from the Simmental GPSM gene lists, we identified minimal tissue-specific expression. We did not identify significant or suggestive tissue enrichments in the Simmental GPSM gene set using the Human Protein Atlas in TissueEnrich. Uterine tissue was the only tissue showing suggestive enrichment.

Pathway analyses of envGWAS candidate gene sets in all three populations pointed towards a role in neural development and signaling in modulating adaptation. We used TSEA in humans and *C. elegans* to provide further evidence of brain and nervous system tissues involvement in environmental adaptation. Using *C. elegans* tissues allowed us to refine the expression of conserved genes to individual neuron resolution. Using gene expression data from the Human Protein Atlas in the TissueEnrich software, we identify the cerebral cortex to be the lone significant tissue among our candidate gene sets from Red Angus and Gelbvieh envGWAS analyses (**S8 and S9 Tables**). These results agreed with TSEA using GTEx data. Despite identifying similar neural pathways in the Simmental population, we did not observe enriched expression in brain tissue in either human TSEA with this gene list.

To further probe the specific brain regions expressing envGWAS candidate genes, we performed a brain-specific expression enrichment analysis using the pSI tool with human brain expression data from BrainSpan and the Allen Brain Atlas [[49]](https://paperpile.com/c/AKVIGO/mn0ji). Complete results are reported in **S10 and S11 Tables.** The only brain region significantly enriched for envGWAS candidate genes was the cortex in Simmental (p = 1.525x10^-4^). Interestingly, Simmental was the only population in which Brain tissue did not show enriched expression in the GTEx data. The envGWAS candidate genes from Red Angus and Gelbvieh showed suggestive enrichments in expression in the Cerebellum (p = 0.096) and Striatum, respectively (p = 0.059). While some suggestive cell-type specific expression differences existed with each gene list, we did not observe any tissues with conserved expression across populations.

**Table A. GPSM and envGWAS gene dropping simulation results.** Ten repetitions of a 200,000 SNP gene dropping experiment through the complete Red Angus pedigree. Real GPSM and envGWAS phenotypes were used to identify significant SNPs (q-value < 0.1). Multiple nearby SNPs were grouped as “genomic regions'' when they were within 1Mb of one another.

| **Analysis** | **Analyzed Individuals** | **Median Number Significant Loci (SD)** | **Median Significant q-value (SD)** |
| --- | --- | --- | --- |
| GPSM | 15,315 | 0.4 (0.52) | 0.047 (0.027) |
| envGWAS | 15,315 | 1.36 (1.56) | 0.051 (0.031) |

**Table B. GPSM datasets from three major U.S. beef cattle populations.** Sample sizes are reported prior to and after filtering on individual call rate individuals with reported birth dates.

| **Breed** | **Sample Size  (After Filtering)** | **Median Birth Date** | **Mean Birth Date** | **Min Birth Date** | **Max Birth Date** |
| --- | --- | --- | --- | --- | --- |
| Red Angus | 16,331 (15,295^1^) | 2014-10-16 | 2013-12-26 | 1975-03-21 | 2017-04-23 |
| Simmental | 17,468 (15,350) | 2013-08-09 | 2011-08-11 | 1966-02-16 | 2016-04-06 |
| Gelbvieh | 12,563 (12,031) | 2015-01-11 | 2013-09-23 | 1970-12-02 | 2016-09-21 |

^1^ After removing non-purebred animals

**Table C. The proportion of variation in birth date explained (PVE) by markers in GPSM analysis.** PVE calculated for each population dataset in full, and subsetted to individuals born within the last 20 or 10 years. The standard errors of PVE estimates are reported in parentheses.

| **Population** | **Full Dataset PVE (se)** | **20-year PVE (se)** | **10-year PVE (se)** | **Shuffled PVE (se)** |
| --- | --- | --- | --- | --- |
| Red Angus | 0.520 (0.013) | 0.358 (0.013) | 0.406 (0.014) | 9.82 x 10^-6^ (0.002) |
| Simmental | 0.588 (0.009) | 0.551 (0.010) | 0.401 (0.014) | 9.70 x 10^-4^ (0.003) |
| Gelbvieh | 0.459 (0.015) | 0.454 (0.015) | 0.361 (0.014) | 5.05 x 10^-4^ (0.004) |

**Table D. Summary statistics of allele frequency change (ΔAF) per generation for significant GPSM SNPs.** ΔAF is the slope of a simple regression of allele frequency on birth date multiplied by a generation interval of 5 years.

| **Breed** | **N SNPs (GPSM q < 0.1)** | **Mean ΔAF per generation (sd)** | **Median ΔAF per generation** | **Min ΔAF per generation** | **Max ΔAF per generation** |
| --- | --- | --- | --- | --- | --- |
| Red Angus | 268 | 0.018 (0.011) | 0.017 | 4.97 x 10^-5^ | 0.076 |
| Simmental | 548 | 0.024 (0.017) | 0.022 | 6.79 x 10^-5^ | 0.093 |
| Gelbvieh | 762 | 0.033 (0.028) | 0.024 | 1.01 x 10^-4^ | 0.223 |

**Table E. Significant GPSM variants identified in at least two populations.** Lead SNPs from significant loci identified in GPSM analyses of Red Angus, Simmental, and Gelbvieh cattle populations. Locus reported if it was identified in GPSM analysis of at least two populations. Candidate gene is the annotated gene closest to lowest q-value SNP in peak if < 200 kb away. Associations are from cattle literature unless otherwise reported.

| **CHR** | **POS** | **Nearest Candidate Gene(s) (Distance)** | **Known Candidate Gene Associations** | **References** | **Datasets** |
| --- | --- | --- | --- | --- | --- |
| 1 | 157,913,264 | LOC112448253 (within) | lncRNA, misplaced BoLA SNPs?, PRDM9 association? |  | ALL |
| 2 | 53,151,541 | *ARHGAP15* (within) | Immune functions, Trypanosomiasis resistance | [[10–12]](https://paperpile.com/c/AKVIGO/InarH+hOSw2+tItrU) | ALL |
| 12 | 46,730,506 | *DACH1* (182.1 kb) | Feed efficiency/growth | [[6]](https://paperpile.com/c/AKVIGO/zHr9Z) | ALL |
| 14 | 59,774,083 | *LRP12* (261.5 kb), LOC112449532 (113.6 kb) | (*LRP12*) Feed efficiency/growth | [[9,50]](https://paperpile.com/c/AKVIGO/zrOFa+6pGRx) | ALL |
| 16 | 955,146 | *ADORA1* (within),  *MYBPH* (2.0 kb) | Fertility/immune (*ADORA1*)/muscle growth (*MYBPH*) | [[13,15,51]](https://paperpile.com/c/AKVIGO/dTsC+Hemd+BsGl) | ALL |
| 23 | 1,768,070 | LOC782044 (25.7 kb) |  |  | ALL |
| 28 | 640,998 | *RHOU* (56.3 kb),  Olfactory gene cluster (166.5kb) | Bone development, Innate immune system | [[52]](https://paperpile.com/c/AKVIGO/yVaZm) | ALL |
| 1 | 3,144,864 | URB1 (within) | Embryonic lethal (pigs) | [[53]](https://paperpile.com/c/AKVIGO/ojBgA) | SIM/GEL |
| 1 | 73,642,609 | > 200 kb to a gene |  |  | SIM/GEL |
| 3 | 119,429,700 | CSF2RA (within) | TB Resistance | [[54]](https://paperpile.com/c/AKVIGO/QYztY) | RAN/SIM |
| 5 | 5,266,489 | ENSBTAG00000050164 (within) | lncRNA |  | SIM/GEL |
| 8 | 113,265,058 | > 200 kb to a gene |  |  | SIM/GEL |
| 9 | 78,704,840 | PWWP2B  (23.6 kb) | Bovine fetus muscle expression | [[55]](https://paperpile.com/c/AKVIGO/ON5ne) | RAN/GEL |
| 9 | 104,228,150 | PDCD2 (111 kb) |  |  | SIM/GEL |
| 10 | 940,793 | MCC (within) |  |  | SIM/GEL |
| 12 | 518,595 | PCDH20 (37.7 kb) |  |  | SIM/GEL |
| 15 | 77,106,772 | DDB2 (within) | Calving Ease | [[56]](https://paperpile.com/c/AKVIGO/J5M8I) | RAN/GEL |
| 15 | 84,680,379 | LOC617614 | Olfactory Receptor |  | SIM/GEL |
| 16 | 78,129,493 | > 200 kb to a gene |  |  | SIM/GEL |
| 19 | 53,947,810 | BIRC5 (within) | RFI (DE), Fertility and Reproduction | [[57]](https://paperpile.com/c/AKVIGO/QNlsU) | RAN/GEL |
| 22 | 24,625,216 | Between CNTN6 and CNTN4 | Known Milk Yield QTL | [[58]](https://paperpile.com/c/AKVIGO/sfkBN) | SIM/GEL |
| 28 | 20,762,645 | > 200 kb to a gene |  |  | SIM/GEL |

**Table F. Ecoregion distribution of Red Angus, Simmental, and Gelbvieh populations.** Counts of analyzed individuals in each region for each dataset after filtering and region assignment based on individual’s breeder zip code.

| **Region** | **Red Angus** | **Simmental** | **Gelbvieh** |
| --- | --- | --- | --- |
| Desert | 367^1^ | 321^1^ | 408^1^ |
| Southeast | 615^1^ | 1,073^1,2^ | 422^1^ |
| High Plains | 2,796^1,2^ | 3,645^1,2^ | 4,022^1,2^ |
| Rainforest | 0 | 62 | 1 |
| Arid Prairie | 1,206^1,2^ | 181 | 87 |
| Foothills | 136 | 0 | 0 |
| Forested Mountains | 4,525^1,2^ | 2,589^1,2^ | 704^1,2^ |
| Fescue Belt | 3,011^1,2^ | 4,393^1,2^ | 4,482^1,2^ |
| Upper Midwest & Northeast | 1,513^1,2^ | 2,524^1,2^ | 1,072^1,2^ |
| **Total** | **14,169** | **14,788** | **11,198** |

^1^ included in multivariate discrete envGWAS analysis

^2^ included in “large region” multivariate envGWAS analysis

**Table G. Univariate REML estimates of PVE for continuous environmental variables in genotyped Red Angus, Simmental, and Gelbvieh populations.** Standard errors for PVE estimates are reported in parentheses.

| **Variable** | **Red Angus PVE (se)** | **Simmental PVE (se)** | **Gelbvieh PVE (se)** |
| --- | --- | --- | --- |
| Temperature | 0.597 (0.010) | 0.586 (0.011) | 0.691 (0.010) |
| Precipitation | 0.526 (0.011) | 0.602 (0.011) | 0.677 (0.010) |
| Elevation | 0.594 (0.010) | 0.585 (0.011) | 0.644 (0.011) |

**Table H. Univariate estimates of PVE for discrete ecoregion assignment in genotyped Red Angus, Simmental, and Gelbvieh populations**. Standard errors for PVE estimates are reported in parentheses.

| **Variable** | **Red Angus PVE (se)** | **Simmental PVE (se)** | **Gelbvieh PVE (se)** |
| --- | --- | --- | --- |
| Desert | 0.646 (0.010) | 0.517 (0.012) | 0.726 (0.010) |
| Southeast (SE) | 0.408 (0.010) | 0.547 (0.013) | 0.478 (0.013) |
| High Plains (HP) | 0.641 (0.011) | 0.588 (0.010) | 0.694 (0.010) |
| Arid Prairie (AP) | 0.463 (0.011) | 0.566 (0.014) | NA |
| Forested Mountains (FM) | 0.575 (0.011) | 0.594 (0.010) | 0.615 (0.013) |
| Fescue Belt (FB) | 0.673 (0.010) | 0.545 (0.011) | 0.649 (0.011) |
| Upper Midwest & Northeast (UMWNE) | 0.548 (0.012) | 0.509 (0.012) | 0.609 (0.013) |

**Table I. Candidate genes for discrete ecoregion multivariate envGWAS in Red Angus cattle.** Lead SNP in envGWAS peak is reported along with nearest plausible candidate genes (provided < 250 kb from lead SNP). If association was also identified in univariate analysis, it is reported. Potentially adaptive associations are reported along with references.

| **CHR** | **POS** | **Nearest Candidate Gene(s) (Distance)** | **Univariate Continuous Association** | **Univariate Ecoregion Association** | **Candidate Gene Adaptive Associations** | **Reference** | **Breed(s)** |
| --- | --- | --- | --- | --- | --- | --- | --- |
| 2 | 7,571,508 | DIRC1 (110.3 kb),  COL5A2 (212.7 kb) | Multivariate, Temperature | FM | Sweep region (human), blood pressure (human) | [[25,59,60]](https://paperpile.com/c/AKVIGO/Yxs7+WtDT+pzfW) | RAN |
| 2 | 25,519,381 | GORASP2 (4.47 kb) | N/A | AP | Adaptive signature (fish) | [[61]](https://paperpile.com/c/AKVIGO/LyFO2) | RAN |
| 4 | 43,928,337 | MAGI2 (within) | N/A | UMWNE | Selection signature, imprinted (cattle) | [[62,63]](https://paperpile.com/c/AKVIGO/BRtJT+DB8MS) | RAN |
| 5 | 59,498,938 | LOC788524 (OR9K2) (1.42 kb) [Olfactory cluster] | N/A | FM | Olfactory receptor cluster, selection signature (bison) | [[20]](https://paperpile.com/c/AKVIGO/Db06R) | RAN |
| 6 | 96,217,506 | RASGEF1B (within) | N/A | HP | Immune function, adaptation signature (human), Response to viral infections. | [[64,65]](https://paperpile.com/c/AKVIGO/6WT8O+CXtVi) | RAN |
| 8 | 84,146,584 | CENPP (within) | N/A | N/A | African cattle CNV, hypoxia (human) | [[66,67]](https://paperpile.com/c/AKVIGO/RdPry+O6SFm) | RAN |
| 12 | 58,438,020 | N/A | N/A | N/A |  |  | RAN |
| 13 | 75,784,164 | ZMYND8 (within) |  |  | Immune function, DNA damage repair | [[68,69]](https://paperpile.com/c/AKVIGO/2wFD9+tW6s4) | RAN |
| 13 | 81,716,869 | PFDN4 (71.97 kb) | N/A | HP | Dermatitis (humans) | [[70]](https://paperpile.com/c/AKVIGO/FTMwp) | RAN |
| 22 | 48,873,180 | DUSP7 (40.83 kb) |  |  | Heat stress/sperm motility (cattle),  Stress response | [[71,72]](https://paperpile.com/c/AKVIGO/hwo0a+y1RTc) | RAN |
| 23 | 1,768,070 |  |  | AP, FM |  |  | RAN |
| 24 | 10,628,280 | CDH7 (151.7 kb) | MV | AP | Developmental processes | [[73]](https://paperpile.com/c/AKVIGO/gBZxI) | RAN |
| 24 | 62,228,300 | LOC100298064 (HAVCR1) (15.2kb) |  | AP | Immune function | [[74]](https://paperpile.com/c/AKVIGO/zAj0v) | RAN |
| 25 | 26,511,450 | SPN (CD43) (within) | N/A | SE | MHC-Class I, Immune response (cattle) | [[75]](https://paperpile.com/c/AKVIGO/droxX) | RAN |
| 25 | 35,085,041 | CUX1 (67.8 kb) | Multivariate | N/A | Hair phenotype (goats, mice) | [[76,77]](https://paperpile.com/c/AKVIGO/iWvCF+phCkf) |  |
| 26 | 34,432,722 | ADRB1 (81.5 kb) | Temperature |  | Selection (sporting dogs), climate adaptation (lizards) | [[78,79]](https://paperpile.com/c/AKVIGO/hj5Fi+2iC0F) | RAN |

**Table J. Candidate genes identified in multivariate envGWAS analyses using continuous environmental attributes as dependent variables.** Chromosome and genomic positions are for lead SNP in peak. Closest gene is identified as a candidate (if < 250 kb from lead SNP).

| **CHR** | **POS** | **Nearest Annotated Gene (Distance)** | **Univariate association**  **(if p < 1×10^-5^)** | **Adaptation- related associations** | **References** | **Breed(s)** |
| --- | --- | --- | --- | --- | --- | --- |
| 2 | 7,571,508 | DIRC1 (110.3 kb), COL5A2 (212.7 kb) | Temperature | Sweep region (human), blood pressure (human) | [[25,59]](https://paperpile.com/c/AKVIGO/Yxs7+WtDT) | RAN |
| 2 | 16,014,918 | No genes < 200 kb | Temperature |  |  | RAN |
| 4 | 32,945,494 | ABCB1 (within) | Temperature | Drug resistance, human adaptation, cattle health traits | [[80–82]](https://paperpile.com/c/AKVIGO/lwG1E+w3DaY+JW9P7) | RAN |
| 5 | 6,551,121 | E2F7 (68.592 kb) |  | Body weight, bone density (human) | [[83,84]](https://paperpile.com/c/AKVIGO/ZD3II+t5rx3) | RAN |
| 6 | 57,392,860 | TBC1D1 (within) |  | Selection (cattle, chickens), body size (chickens, mice), immune traits (lymphocytes, etc.), obesity (humans) | [[18,85–90]](https://paperpile.com/c/AKVIGO/4V5fB+vXEPE+MPJFh+5Fmi2+6zRNN+HTKAR+LrNMd) | RAN |
| 7 | 106,527,874 | EFNA5 (within) | Precipitation |  |  | RAN |
| 10 | 69,692,497 | AP5M1 (within) |  | Selection (humans) | [[91]](https://paperpile.com/c/AKVIGO/aGylD) | RAN |
| 10 | 84,307,302 | DPF3 (within) | Precipitation |  |  | RAN |
| 15 | 2,232,970 | GRIA4 (within) | Elevation | Cold tolerance (cattle) | [[92]](https://paperpile.com/c/AKVIGO/pzgyW) | RAN |
| 15 | 71,402,156 | LRRC4C (within) |  | Altitude adaptation (humans) | [[93]](https://paperpile.com/c/AKVIGO/1DvZn) | RAN |
| 22 | 4,815,225 | RBMS3 (156.7 kb) | Precipitation | Tropical adaptation (humans),  Sweep region (humans),  Pleiotropic QTL (cattle) | [[25,94,95]](https://paperpile.com/c/AKVIGO/KS7er+Yxs7+1WICU) | RAN |
| 22 | 5,297,061 | GADL1 (within) |  | Associated with climate variables in Mediterranean cattle, blood metabolites, human adaptation | [[96–98]](https://paperpile.com/c/AKVIGO/y5XwM+1CxXG+XHQJY) | RAN |
| 24 | 10,628,280 | CDH7 (150.58 kb) |  |  |  | RAN |
| 24 | 35,718,635 | ADCYAP1 (14.3 kb) |  | Circadian rhythm (birds), chronotype (humans) | [[99–101]](https://paperpile.com/c/AKVIGO/d8YE2+fejtT+HMCNO) | RAN |
| 25 | 35,085,041 | CUX1 (67.8 kb) |  | Hair phenotypes (goats, mice) | [[76,77]](https://paperpile.com/c/AKVIGO/iWvCF+phCkf) | RAN |
| 25 | 39,464,325 | LOC101904513 | Precipitation | ncRNA |  | RAN |
| 28 | 28,979,090 | PLA2G12B (16.0 kb) | Temperature and Elevation | Local adaptation (humans) | [[102,103]](https://paperpile.com/c/AKVIGO/7BOtE+XnlxC) | RAN |
| 29 | 44,555,972 | BBS1 (within) |  | Obesity, energy homeostasis (human) | [[104–106]](https://paperpile.com/c/AKVIGO/wVJtq+0yzxR+8QMUk) | RAN |
| 1 | 26,621,249 | ROBO1 (within) |  | Neuron development (dogs, cattle, pigs), selection signature (cattle), sporting dogs, temperature acclimation (pigs) | [[22,63,78,107]](https://paperpile.com/c/AKVIGO/hj5Fi+DB8MS+I05mt+XrRwZ) | SIM |
| 1 | 134,777,473 | CEP63 (within) |  | Height (human), thermal adaptation (fish) | [[108,109]](https://paperpile.com/c/AKVIGO/9e36O+tSPIP) | SIM |
| 3 | 2,966,062 | UCK2 (18.65 kb) |  | Osmoregulation (fish), disease response (cattle) | [[110,111]](https://paperpile.com/c/AKVIGO/PFema+zO8Gr) | SIM |
| 8 | 64,286,414 | ENSBTAG00000054262 |  | lncRNA |  | SIM |
| 10 | 16,366,200 | KIF23 (24.83 kb) |  | Hepatic function (cattle) | [[112,113]](https://paperpile.com/c/AKVIGO/QpuWq+DJea4) | SIM |
| 17 | 51,685,973 | LOC100847522 (88.07 kb) |  | ncRNA |  | SIM |
| 20 | 54,365,387 |  | Precipitation, Elevation |  |  | SIM |
| 23 | 1,768,070 | LOC782044 | Precipitation, Elevation |  |  | SIM |
| 26 | 34,125,126 | NRAP (within) |  | Meat traits (cattle), under selection in Eastern Finncattle, neuron development | [[114–116]](https://paperpile.com/c/AKVIGO/ZFGc9+1Xem6+xCuPV) | SIM |
| 28 | 640,998 | RHOU (56.34 kb) | Elevation |  |  | SIM |
| 29 | 36,766,544 | LOC112444895 (137.67 kb) | Precipitation, Elevation | ncRNA |  | SIM |
| 2 | 116,117,760 | SPHKAP (within) | Elevation | Insulin secretion, kidney disease susceptibility (human) | [[117,118]](https://paperpile.com/c/AKVIGO/80XBt+7FSno) | GEL |
| 3 | 65,627,833 | ADGRL4 (39.34 kb) | Temperature |  |  | GEL |
| 4 | 35,457,854 | SEMA3D (within) |  | Calving ease, neuron/axon guidance | [[119–121]](https://paperpile.com/c/AKVIGO/bdycD+UWugu+7sCyQ) | GEL |
| 4 | 95,515,907 | LOC785077 (30.81 kb) | Precipitation |  |  | GEL |
| 11 | 81,911,781 | FAM49A (26.09 kb) | Temperature |  |  | GEL |
| 14 | 73,663,377 | CALB1 (within) | Elevation | Selection signature (pigs) | [[122]](https://paperpile.com/c/AKVIGO/Ss1pk) | GEL |
| 23 | 1,760,296 | LOC782044 (17.22 kb) |  |  |  | GEL |
| 25 | 663,051 | LOC531296 (within)/MSLN | Temperature | Feed intake (cattle) | [[57]](https://paperpile.com/c/AKVIGO/QNlsU) | GEL |

**References**

1. [Berg IL, Neumann R, Lam K-WG, Sarbajna S, Odenthal-Hesse L, May CA, et al. PRDM9 variation strongly influences recombination hot-spot activity and meiotic instability in humans. Nat Genet. 2010;42: 859–863.](http://paperpile.com/b/AKVIGO/obSIr)

2. [Baudat F, Buard J, Grey C, Fledel-Alon A, Ober C, Przeworski M, et al. PRDM9 is a major determinant of meiotic recombination hotspots in humans and mice. Science. 2010;327: 836–840.](http://paperpile.com/b/AKVIGO/XPXM6)

3. [Ma L, O’Connell JR, VanRaden PM, Shen B, Padhi A, Sun C, et al. Cattle Sex-Specific Recombination and Genetic Control from a Large Pedigree Analysis. PLoS Genet. 2015;11: e1005387.](http://paperpile.com/b/AKVIGO/dEvkm)

4. [Gonen S, Battagin M, Johnston SE, Gorjanc G, Hickey JM. The potential of shifting recombination hotspots to increase genetic gain in livestock breeding. Genet Sel Evol. 2017;49: 55.](http://paperpile.com/b/AKVIGO/BRGnr)

5. [Kuehn LA, Thallman RM. Across-Breed EPD Tables For The Year 2016 Adjusted To Breed Differences For Birth Year Of 2014. 2016 [cited 9 Feb 2020]. Available:](http://paperpile.com/b/AKVIGO/zicE8) <https://digitalcommons.unl.edu/hruskareports/380/>

6. [Serão NV, González-Peña D, Beever JE, Faulkner DB, Southey BR, Rodriguez-Zas SL. Single nucleotide polymorphisms and haplotypes associated with feed efficiency in beef cattle. BMC Genet. 2013;14: 94.](http://paperpile.com/b/AKVIGO/zHr9Z)

7. [Snelling WM, Allan MF, Keele JW, Kuehn LA, McDaneld T, Smith TPL, et al. Genome-wide association study of growth in crossbred beef cattle. J Anim Sci. 2010;88: 837–848.](http://paperpile.com/b/AKVIGO/uSWrT)

8. [Mateescu RG, Garrick DJ, Reecy JM. Network Analysis Reveals Putative Genes Affecting Meat Quality in Angus Cattle. Front Genet. 2017;8: 171.](http://paperpile.com/b/AKVIGO/d67MT)

9. [Seabury CM, Oldeschulte DL, Saatchi M, Beever JE, Decker JE, Halley YA, et al. Genome-wide association study for feed efficiency and growth traits in U.S. beef cattle. BMC Genomics. 2017;18: 386.](http://paperpile.com/b/AKVIGO/zrOFa)

10. [Noyes H, Brass A, Obara I, Anderson S, Archibald AL, Bradley DG, et al. Genetic and expression analysis of cattle identifies candidate genes in pathways responding to Trypanosoma congolense infection. Proc Natl Acad Sci U S A. 2011;108: 9304–9309.](http://paperpile.com/b/AKVIGO/InarH)

11. [Smetko A, Soudre A, Silbermayr K, Müller S, Brem G, Hanotte O, et al. Trypanosomosis: potential driver of selection in African cattle. Front Genet. 2015;6: 137.](http://paperpile.com/b/AKVIGO/hOSw2)

12. [Álvarez I, Pérez-Pardal L, Traoré A, Fernández I, Goyache F. African Cattle do not Carry Unique Mutations on the Exon 9 of the ARHGAP15 Gene. Anim Biotechnol. 2016;27: 9–12.](http://paperpile.com/b/AKVIGO/tItrU)

13. [Seo J, Osorio JS, Loor JJ. Purinergic signaling gene network expression in bovine polymorphonuclear neutrophils during the peripartal period. J Dairy Sci. 2013;96: 7675–7683.](http://paperpile.com/b/AKVIGO/dTsC)

14. [Mediero A, Cronstein BN. Adenosine and bone metabolism. Trends Endocrinol Metab. 2013;24: 290–300.](http://paperpile.com/b/AKVIGO/y8Bfi)

15. [Chelh I, Picard B, Hocquette J-F, Cassar-Malek I. Myostatin inactivation induces a similar muscle molecular signature in double-muscled cattle as in mice. Animal. 2011;5: 278–286.](http://paperpile.com/b/AKVIGO/Hemd)

16. [Weber KL, Welly BT, Van Eenennaam AL, Young AE, Porto-Neto LR, Reverter A, et al. Identification of Gene Networks for Residual Feed Intake in Angus Cattle Using Genomic Prediction and RNA-seq. PLoS One. 2016;11: e0152274.](http://paperpile.com/b/AKVIGO/Bv6Ay)

17. [Alexandre PA, Kogelman LJA, Santana MHA, Passarelli D, Pulz LH, Fantinato-Neto P, et al. Liver transcriptomic networks reveal main biological processes associated with feed efficiency in beef cattle. BMC Genomics. 2015;16: 1073.](http://paperpile.com/b/AKVIGO/xXhXu)

18. [Zhao F, McParland S, Kearney F, Du L, Berry DP. Detection of selection signatures in dairy and beef cattle using high-density genomic information. Genet Sel Evol. 2015;47: 49.](http://paperpile.com/b/AKVIGO/4V5fB)

19. [Li M, Tian S, Jin L, Zhou G, Li Y, Zhang Y, et al. Genomic analyses identify distinct patterns of selection in domesticated pigs and Tibetan wild boars. Nat Genet. 2013;45: 1431–1438.](http://paperpile.com/b/AKVIGO/3uWbY)

20. [Gautier M, Moazami-Goudarzi K, Levéziel H, Parinello H, Grohs C, Rialle S, et al. Deciphering the Wisent Demographic and Adaptive Histories from Individual Whole-Genome Sequences. Mol Biol Evol. 2016;33: 2801–2814.](http://paperpile.com/b/AKVIGO/Db06R)

21. [Magalhães AFB, de Camargo GMF, Fernandes GA Junior, Gordo DGM, Tonussi RL, Costa RB, et al. Genome-Wide Association Study of Meat Quality Traits in Nellore Cattle. PLoS One. 2016;11: e0157845.](http://paperpile.com/b/AKVIGO/UeBVu)

22. [Ai H, Fang X, Yang B, Huang Z, Chen H, Mao L, et al. Adaptation and possible ancient interspecies introgression in pigs identified by whole-genome sequencing. Nat Genet. 2015;47: 217–225.](http://paperpile.com/b/AKVIGO/XrRwZ)

23. [Boulant JA, Dean JB. Temperature receptors in the central nervous system. Annu Rev Physiol. 1986;48: 639–654.](http://paperpile.com/b/AKVIGO/aUEvi)

24. [Mombaerts P. Axonal wiring in the mouse olfactory system. Annu Rev Cell Dev Biol. 2006;22: 713–737.](http://paperpile.com/b/AKVIGO/xEqbM)

25. [Williamson SH, Hubisz MJ, Clark AG, Payseur BA, Bustamante CD, Nielsen R. Localizing recent adaptive evolution in the human genome. PLoS Genet. 2007;3: e90.](http://paperpile.com/b/AKVIGO/Yxs7)

26. [Kellogg DL Jr, Pérgola PE, Piest KL, Kosiba WA, Crandall CG, Grossmann M, et al. Cutaneous active vasodilation in humans is mediated by cholinergic nerve cotransmission. Circ Res. 1995;77: 1222–1228.](http://paperpile.com/b/AKVIGO/3IdfL)

27. [Kellogg DL Jr, Hodges GJ, Orozco CR, Phillips TM, Zhao JL, Johnson JM. Cholinergic mechanisms of cutaneous active vasodilation during heat stress in cystic fibrosis. J Appl Physiol. 2007;103: 963–968.](http://paperpile.com/b/AKVIGO/MH9ju)

28. [Joyner MJ, Dietz NM. Sympathetic vasodilation in human muscle. Acta Physiol Scand. 2003;177: 329–336.](http://paperpile.com/b/AKVIGO/8S1Hj)

29. [Smith CJ, Johnson JM. Responses to hyperthermia. Optimizing heat dissipation by convection and evaporation: Neural control of skin blood flow and sweating in humans. Auton Neurosci. 2016;196: 25–36.](http://paperpile.com/b/AKVIGO/4IZKu)

30. [Takemoto Y. Amino acids that centrally influence blood pressure and regional blood flow in conscious rats. J Amino Acids. 2012;2012: 831759.](http://paperpile.com/b/AKVIGO/ddKIO)

31. [Meng W, Tobin JR, Busija DW. Glutamate-induced cerebral vasodilation is mediated by nitric oxide through N-methyl-D-aspartate receptors. Stroke. 1995;26: 857–62; discussion 863.](http://paperpile.com/b/AKVIGO/9nYyW)

32. [Braz CU, Rowan TN, Schnabel RD, Decker JE. Extensive genome-wide association analyses identify genotype-by-environment interactions of growth traits in Simmental cattle. doi:](http://paperpile.com/b/AKVIGO/z7Wql)[10.1101/2020.01.09.900902](http://dx.doi.org/10.1101/2020.01.09.900902)

33. [Morrison SF. Central control of body temperature. F1000Res. 2016;5. doi:](http://paperpile.com/b/AKVIGO/mHGXG)[10.12688/f1000research.7958.1](http://dx.doi.org/10.12688/f1000research.7958.1)

34. [Conrad KP. Unveiling the vasodilatory actions and mechanisms of relaxin. Hypertension. 2010;56: 2–9.](http://paperpile.com/b/AKVIGO/ytYW3)

35. [Daanen HAM, Van Marken Lichtenbelt WD. Human whole body cold adaptation. Temperature (Austin). 2016;3: 104–118.](http://paperpile.com/b/AKVIGO/rJYEt)

36. [Choshniak I, McEwan-Jenkinson D, Blatchford DR, Peaker M. Blood flow and catecholamine concentration in bovine and caprine skin during thermal sweating. Comp Biochem Physiol C. 1982;71C: 37–42.](http://paperpile.com/b/AKVIGO/kQDQC)

37. [Garner JB, Douglas ML, Williams SRO, Wales WJ, Marett LC, Nguyen TTT, et al. Genomic Selection Improves Heat Tolerance in Dairy Cattle. Sci Rep. 2016;6: 34114.](http://paperpile.com/b/AKVIGO/Lu4mA)

38. [Rhoads ML, Rhoads RP, VanBaale MJ, Collier RJ, Sanders SR, Weber WJ, et al. Effects of heat stress and plane of nutrition on lactating Holstein cows: I. Production, metabolism, and aspects of circulating somatotropin. J Dairy Sci. 2009;92: 1986–1997.](http://paperpile.com/b/AKVIGO/CFRWj)

39. [El-Nouty FD, Elbanna IM, Davis TP, Johnson HD. Aldosterone and ADH response to heat and dehydration in cattle. J Appl Physiol. 1980;48: 249–255.](http://paperpile.com/b/AKVIGO/nTNfj)

40. [Itoh F, Obara Y, Rose MT, Fuse H, Hashimoto H. Insulin and Glucagon Secretion in Lactating Cows During Heat Exposure1. Available:](http://paperpile.com/b/AKVIGO/1wF6W) <https://academic.oup.com/jas/article-abstract/76/8/2182/4643238>

41. [Sanz Fernandez MV, Stoakes SK, Abuajamieh M, Seibert JT, Johnson JS, Horst EA, et al. Heat stress increases insulin sensitivity in pigs. Physiol Rep. 2015;3. doi:](http://paperpile.com/b/AKVIGO/tZSd5)[10.14814/phy2.12478](http://dx.doi.org/10.14814/phy2.12478)

42. [Keogh K, Kenny DA, Kelly AK, Waters SM. Insulin secretion and signaling in response to dietary restriction and subsequent re-alimentation in cattle. Physiol Genomics. 2015;47: 344–354.](http://paperpile.com/b/AKVIGO/l6JCI)

43. [Tesmer LA, Lundy SK, Sarkar S, Fox DA. Th17 cells in human disease. Immunol Rev. 2008;223: 87–113.](http://paperpile.com/b/AKVIGO/JhZ8Q)

44. [Romagnani S. T-cell subsets (Th1 versus Th2). Ann Allergy Asthma Immunol. 2000;85: 9–18; quiz 18, 21.](http://paperpile.com/b/AKVIGO/WxSKH)

45. [Deschamps M, Laval G, Fagny M, Itan Y, Abel L, Casanova J-L, et al. Genomic Signatures of Selective Pressures and Introgression from Archaic Hominins at Human Innate Immunity Genes. Am J Hum Genet. 2016;98: 5–21.](http://paperpile.com/b/AKVIGO/Wcnik)

46. [Crawford JE, Amaru R, Song J, Julian CG, Racimo F, Cheng JY, et al. Natural Selection on Genes Related to Cardiovascular Health in High-Altitude Adapted Andeans. Am J Hum Genet. 2017;101: 752–767.](http://paperpile.com/b/AKVIGO/08tFh)

47. [The GTEx Consortium. The Genotype-Tissue Expression (GTEx) pilot analysis: Multitissue gene regulation in humans. Science. 2015;348: 648–660.](http://paperpile.com/b/AKVIGO/vc9jV)

48. [Xu X, Wells AB, O’Brien DR, Nehorai A, Dougherty JD. Cell type-specific expression analysis to identify putative cellular mechanisms for neurogenetic disorders. J Neurosci. 2014;34: 1420–1431.](http://paperpile.com/b/AKVIGO/j9rRl)

49. [Sunkin SM, Ng L, Lau C, Dolbeare T, Gilbert TL, Thompson CL, et al. Allen Brain Atlas: an integrated spatio-temporal portal for exploring the central nervous system. Nucleic Acids Res. 2013;41: D996–D1008.](http://paperpile.com/b/AKVIGO/mn0ji)

50. [Müller M-P, -P. Müller M, Rothammer S, Seichter D, Russ I, Hinrichs D, et al. Genome-wide mapping of 10 calving and fertility traits in Holstein dairy cattle with special regard to chromosome 18. Journal of Dairy Science. 2017. pp. 1987–2006. doi:](http://paperpile.com/b/AKVIGO/6pGRx)[10.3168/jds.2016-11506](http://dx.doi.org/10.3168/jds.2016-11506)

51. [Seo J, Osorio JS, Schmitt E, Corrêa MN, Bertoni G, Trevisi E, et al. Hepatic purinergic signaling gene network expression and its relationship with inflammation and oxidative stress biomarkers in blood from peripartal dairy cattle. J Dairy Sci. 2014;97: 861–873.](http://paperpile.com/b/AKVIGO/BsGl)

52. [Espigolan R, Baldi F, Boligon AA, Souza FRP, Fernandes Júnior GA, Gordo DGM, et al. Associations between single nucleotide polymorphisms and carcass traits in Nellore cattle using high-density panels. Genet Mol Res. 2015;14: 11133–11144.](http://paperpile.com/b/AKVIGO/yVaZm)

53. [Derks MFL, Gjuvsland AB, Bosse M, Lopes MS, van Son M, Harlizius B, et al. Loss of function mutations in essential genes cause embryonic lethality in pigs. PLoS Genet. 2019;15: e1008055.](http://paperpile.com/b/AKVIGO/ojBgA)

54. [Meade KG, Gormley E, O’Farrelly C, Park SD, Costello E, Keane J, et al. Antigen stimulation of peripheral blood mononuclear cells from Mycobacterium bovis infected cattle yields evidence for a novel gene expression program. BMC Genomics. 2008;9: 447.](http://paperpile.com/b/AKVIGO/QYztY)

55. [Cassar-Malek I, Boby C, Picard B, Reverter A, Hudson NJ. Molecular regulation of high muscle mass in developing Blonde d’Aquitaine cattle foetuses. Biol Open. 2017;6: 1483–1492.](http://paperpile.com/b/AKVIGO/ON5ne)

56. [Höglund JK, Guldbrandtsen B, Lund MS, Sahana G. Analyzes of genome-wide association follow-up study for calving traits in dairy cattle. BMC Genet. 2012;13: 71.](http://paperpile.com/b/AKVIGO/J5M8I)

57. [Chen Y, Gondro C, Quinn K, Herd RM, Parnell PF, Vanselow B. Global gene expression profiling reveals genes expressed differentially in cattle with high and low residual feed intake. Anim Genet. 2011;42: 475–490.](http://paperpile.com/b/AKVIGO/QNlsU)

58. [Marete AG, Guldbrandtsen B, Lund MS, Fritz S, Sahana G, Boichard D. A Meta-Analysis Including Pre-selected Sequence Variants Associated With Seven Traits in Three French Dairy Cattle Populations. Front Genet. 2018;9: 522.](http://paperpile.com/b/AKVIGO/sfkBN)

59. [Evangelou E, Warren HR, Mosen-Ansorena D, Mifsud B, Pazoki R, Gao H, et al. Genetic analysis of over 1 million people identifies 535 new loci associated with blood pressure traits. Nat Genet. 2018;50: 1412–1425.](http://paperpile.com/b/AKVIGO/WtDT)

60. [Hussin J, Nadeau P, Lefebvre J-F, Labuda D. Haplotype allelic classes for detecting ongoing positive selection. BMC Bioinformatics. 2010;11: 65.](http://paperpile.com/b/AKVIGO/pzfW)

61. [Wang G, Yang E, Smith KJ, Zeng Y, Ji G, Connon R, et al. Gene expression responses of threespine stickleback to salinity: implications for salt-sensitive hypertension. Front Genet. 2014;5: 312.](http://paperpile.com/b/AKVIGO/LyFO2)

62. [Barbaux S, Gascoin-Lachambre G, Buffat C, Monnier P, Mondon F, Tonanny M-B, et al. A genome-wide approach reveals novel imprinted genes expressed in the human placenta. Epigenetics. 2012;7: 1079–1090.](http://paperpile.com/b/AKVIGO/BRtJT)

63. [Boitard S, Boussaha M, Capitan A, Rocha D, Servin B. Uncovering Adaptation from Sequence Data: Lessons from Genome Resequencing of Four Cattle Breeds. Genetics. 2016;203: 433–450.](http://paperpile.com/b/AKVIGO/DB8MS)

64. [Andrade WA, Silva AM, Alves VS, Salgado APC, Melo MB, Andrade HM, et al. Early endosome localization and activity of RasGEF1b, a toll-like receptor-inducible Ras guanine-nucleotide exchange factor. Genes Immun. 2010;11: 447–457.](http://paperpile.com/b/AKVIGO/6WT8O)

65. [Lopez M, Choin J, Sikora M, Siddle K, Harmant C, Costa HA, et al. Genomic Evidence for Local Adaptation of Hunter-Gatherers to the African Rainforest. Curr Biol. 2019;29: 2926–2935.e4.](http://paperpile.com/b/AKVIGO/CXtVi)

66. [Wang MD, Dzama K, Hefer CA, Muchadeyi FC. Genomic population structure and prevalence of copy number variations in South African Nguni cattle. BMC Genomics. 2015;16: 894.](http://paperpile.com/b/AKVIGO/RdPry)

67. [Alkorta-Aranburu G, Beall CM, Witonsky DB, Gebremedhin A, Pritchard JK, Di Rienzo A. The genetic architecture of adaptations to high altitude in Ethiopia. PLoS Genet. 2012;8: e1003110.](http://paperpile.com/b/AKVIGO/O6SFm)

68. [Delgado-Benito V, Rosen DB, Wang Q, Gazumyan A, Pai JA, Oliveira TY, et al. The Chromatin Reader ZMYND8 Regulates Igh Enhancers to Promote Immunoglobulin Class Switch Recombination. Mol Cell. 2018;72: 636–649.e8.](http://paperpile.com/b/AKVIGO/2wFD9)

69. [Gong F, Chiu L-Y, Cox B, Aymard F, Clouaire T, Leung JW, et al. Screen identifies bromodomain protein ZMYND8 in chromatin recognition of transcription-associated DNA damage that promotes homologous recombination. Genes Dev. 2015;29: 197–211.](http://paperpile.com/b/AKVIGO/tW6s4)

70. [Hirota T, Takahashi A, Kubo M, Tsunoda T, Tomita K, Sakashita M, et al. Genome-wide association study identifies eight new susceptibility loci for atopic dermatitis in the Japanese population. Nat Genet. 2012;44: 1222–1226.](http://paperpile.com/b/AKVIGO/FTMwp)

71. [Srikanth K, Kwon A, Lee E, Chung H. Characterization of genes and pathways that respond to heat stress in Holstein calves through transcriptome analysis. Cell Stress Chaperones. 2017;22: 29–42.](http://paperpile.com/b/AKVIGO/hwo0a)

72. [Rahman MB, Kamal MM, Rijsselaere T, Vandaele L, Shamsuddin M, Van Soom A. Altered chromatin condensation of heat-stressed spermatozoa perturbs the dynamics of DNA methylation reprogramming in the paternal genome after in vitro fertilisation in cattle. Reprod Fertil Dev. 2014;26: 1107–1116.](http://paperpile.com/b/AKVIGO/y1RTc)

73. [Aramaki M, Kimura T, Udaka T, Kosaki R, Mitsuhashi T, Okada Y, et al. Embryonic expression profile of chicken CHD7, the ortholog of the causative gene for CHARGE syndrome. Birth Defects Res A Clin Mol Teratol. 2007;79: 50–57.](http://paperpile.com/b/AKVIGO/gBZxI)

74. [Nakajima T, Wooding S, Satta Y, Jinnai N, Goto S, Hayasaka I, et al. Evidence for natural selection in the HAVCR1 gene: high degree of amino-acid variability in the mucin domain of human HAVCR1 protein. Genes Immun. 2005;6: 398–406.](http://paperpile.com/b/AKVIGO/zAj0v)

75. [McLoughlin KE, Nalpas NC, Rue-Albrecht K, Browne JA, Magee DA, Killick KE, et al. RNA-seq Transcriptional Profiling of Peripheral Blood Leukocytes from Cattle Infected with Mycobacterium bovis. Front Immunol. 2014;5: 396.](http://paperpile.com/b/AKVIGO/droxX)

76. [Bertolini F, Servin B, Talenti A, Rochat E, Kim ES, Oget C, et al. Signatures of selection and environmental adaptation across the goat genome post-domestication. Genet Sel Evol. 2018;50: 57.](http://paperpile.com/b/AKVIGO/iWvCF)

77. [Sansregret L, Nepveu A. The multiple roles of CUX1: insights from mouse models and cell-based assays. Gene. 2008;412: 84–94.](http://paperpile.com/b/AKVIGO/phCkf)

78. [Kim J, Williams FJ, Dreger DL, Plassais J, Davis BW, Parker HG, et al. Genetic selection of athletic success in sport-hunting dogs. Proc Natl Acad Sci U S A. 2018;115: E7212–E7221.](http://paperpile.com/b/AKVIGO/hj5Fi)

79. [Rodríguez A, Rusciano T, Hamilton R, Holmes L, Jordan D, Wollenberg Valero KC. Genomic and phenotypic signatures of climate adaptation in an Anolis lizard. Ecol Evol. 2017;7: 6390–6403.](http://paperpile.com/b/AKVIGO/2iC0F)

80. [Wang H, Ding K, Zhang Y, Jin L, Kullo IJ, He F. Comparative and evolutionary pharmacogenetics of ABCB1: complex signatures of positive selection on coding and regulatory regions. Pharmacogenet Genomics. 2007;17: 667–678.](http://paperpile.com/b/AKVIGO/lwG1E)

81. [Wang Z, Wang J, Tantoso E, Wang B, Tai AYP, Ooi LLPJ, et al. Signatures of recent positive selection at the ATP-binding cassette drug transporter superfamily gene loci. Hum Mol Genet. 2007;16: 1367–1380.](http://paperpile.com/b/AKVIGO/w3DaY)

82. [López Herráez D, Bauchet M, Tang K, Theunert C, Pugach I, Li J, et al. Genetic variation and recent positive selection in worldwide human populations: evidence from nearly 1 million SNPs. PLoS One. 2009;4: e7888.](http://paperpile.com/b/AKVIGO/JW9P7)

83. [Kichaev G, Bhatia G, Loh P-R, Gazal S, Burch K, Freund MK, et al. Leveraging Polygenic Functional Enrichment to Improve GWAS Power. Am J Hum Genet. 2019;104: 65–75.](http://paperpile.com/b/AKVIGO/ZD3II)

84. [Tachmazidou I, Süveges D, Min JL, Ritchie GRS, Steinberg J, Walter K, et al. Whole-Genome Sequencing Coupled to Imputation Discovers Genetic Signals for Anthropometric Traits. Am J Hum Genet. 2017;100: 865–884.](http://paperpile.com/b/AKVIGO/t5rx3)

85. [Cardoso DF, de Albuquerque LG, Reimer C, Qanbari S, Erbe M, do Nascimento AV, et al. Genome-wide scan reveals population stratification and footprints of recent selection in Nelore cattle. Genet Sel Evol. 2018;50: 22.](http://paperpile.com/b/AKVIGO/vXEPE)

86. [Rubin C-J, Zody MC, Eriksson J, Meadows JRS, Sherwood E, Webster MT, et al. Whole-genome resequencing reveals loci under selection during chicken domestication. Nature. 2010;464: 587–591.](http://paperpile.com/b/AKVIGO/MPJFh)

87. [Stone S, Abkevich V, Russell DL, Riley R, Timms K, Tran T, et al. TBC1D1 is a candidate for a severe obesity gene and evidence for a gene/gene interaction in obesity predisposition. Hum Mol Genet. 2006;15: 2709–2720.](http://paperpile.com/b/AKVIGO/5Fmi2)

88. [Chadt A, Leicht K, Deshmukh A, Jiang LQ, Scherneck S, Bernhardt U, et al. Tbc1d1 mutation in lean mouse strain confers leanness and protects from diet-induced obesity. Nat Genet. 2008;40: 1354–1359.](http://paperpile.com/b/AKVIGO/6zRNN)

89. [Astle WJ, Elding H, Jiang T, Allen D, Ruklisa D, Mann AL, et al. The Allelic Landscape of Human Blood Cell Trait Variation and Links to Common Complex Disease. Cell. 2016;167: 1415–1429.e19.](http://paperpile.com/b/AKVIGO/HTKAR)

90. [Northrup JM, Shafer ABA, Anderson CR, Coltman DW, Wittemyer G. Fine-scale genetic correlates to condition and migration in a wild cervid. Evol Appl. 2014;7: 937–948.](http://paperpile.com/b/AKVIGO/LrNMd)

91. [Duforet-Frebourg N, Bazin E, Blum MGB. Genome scans for detecting footprints of local adaptation using a Bayesian factor model. Mol Biol Evol. 2014;31: 2483–2495.](http://paperpile.com/b/AKVIGO/aGylD)

92. [Igoshin AV, Yurchenko AA, Belonogova NM, Petrovsky DV, Aitnazarov RB, Soloshenko VA, et al. Genome-wide association study and scan for signatures of selection point to candidate genes for body temperature maintenance under the cold stress in Siberian cattle populations. BMC Genet. 2019;20: 26.](http://paperpile.com/b/AKVIGO/pzgyW)

93. [Wang B, Zhang Y-B, Zhang F, Lin H, Wang X, Wan N, et al. On the origin of Tibetans and their genetic basis in adapting high-altitude environments. PLoS One. 2011;6: e17002.](http://paperpile.com/b/AKVIGO/1DvZn)

94. [Amorim CEG, Daub JT, Salzano FM, Foll M, Excoffier L. Detection of convergent genome-wide signals of adaptation to tropical forests in humans. PLoS One. 2015;10: e0121557.](http://paperpile.com/b/AKVIGO/KS7er)

95. [Granka JM, Henn BM, Gignoux CR, Kidd JM, Bustamante CD, Feldman MW. Limited evidence for classic selective sweeps in African populations. Genetics. 2012;192: 1049–1064.](http://paperpile.com/b/AKVIGO/1WICU)

96. [Flori L, Moazami-Goudarzi K, Alary V, Araba A, Boujenane I, Boushaba N, et al. A genomic map of climate adaptation in Mediterranean cattle breeds. Mol Ecol. 2019;28: 1009–1029.](http://paperpile.com/b/AKVIGO/y5XwM)

97. [Shin S-Y, Fauman EB, Petersen A-K, Krumsiek J, Santos R, Huang J, et al. An atlas of genetic influences on human blood metabolites. Nat Genet. 2014;46: 543–550.](http://paperpile.com/b/AKVIGO/1CxXG)

98. [Key FM, Fu Q, Romagné F, Lachmann M, Andrés AM. Human adaptation and population differentiation in the light of ancient genomes. Nat Commun. 2016;7: 10775.](http://paperpile.com/b/AKVIGO/XHQJY)

99. [Mueller JC, Pulido F, Kempenaers B. Identification of a gene associated with avian migratory behaviour. Proc Biol Sci. 2011;278: 2848–2856.](http://paperpile.com/b/AKVIGO/d8YE2)

100. [Bazzi G, Galimberti A, Hays QR, Bruni I, Cecere JG, Gianfranceschi L, et al. Adcyap1 polymorphism covaries with breeding latitude in a Nearctic migratory songbird, the Wilson’s warbler (Cardellina pusilla). Ecol Evol. 2016;6: 3226–3239.](http://paperpile.com/b/AKVIGO/fejtT)

101. [Jones SE, Lane JM, Wood AR, van Hees VT, Tyrrell J, Beaumont RN, et al. Genome-wide association analyses of chronotype in 697,828 individuals provides insights into circadian rhythms. Nat Commun. 2019;10: 343.](http://paperpile.com/b/AKVIGO/HMCNO)

102. [Sjöstrand AE, Sjödin P, Jakobsson M. Private haplotypes can reveal local adaptation. BMC Genet. 2014;15: 61.](http://paperpile.com/b/AKVIGO/7BOtE)

103. [Pemberton TJ, Absher D, Feldman MW, Myers RM, Rosenberg NA, Li JZ. Genomic patterns of homozygosity in worldwide human populations. Am J Hum Genet. 2012;91: 275–292.](http://paperpile.com/b/AKVIGO/XnlxC)

104. [Guo D-F, Cui H, Zhang Q, Morgan DA, Thedens DR, Nishimura D, et al. The BBSome Controls Energy Homeostasis by Mediating the Transport of the Leptin Receptor to the Plasma Membrane. PLoS Genet. 2016;12: e1005890.](http://paperpile.com/b/AKVIGO/wVJtq)

105. [Rouabhi M, Guo DF, Rahmouni K. Bardet-Biedl Syndrome 1 Gene in the Ventromedial Hypothalamus is Required for Energy Homeostasis. The FASEB Journal. 2016;30: 750.4–750.4.](http://paperpile.com/b/AKVIGO/0yzxR)

106. [Davis RE, Swiderski RE, Rahmouni K, Nishimura DY, Mullins RF, Agassandian K, et al. A knockin mouse model of the Bardet–Biedl syndrome 1 M390R mutation has cilia defects, ventriculomegaly, retinopathy, and obesity. Proc Natl Acad Sci U S A. 2007;104: 19422–19427.](http://paperpile.com/b/AKVIGO/8QMUk)

107. [Dickinson RE, Duncan WC. The SLIT-ROBO pathway: a regulator of cell function with implications for the reproductive system. Reproduction. 2010;139: 697–704.](http://paperpile.com/b/AKVIGO/I05mt)

108. [Wu D-D, Zhang Y-P. Positive selection drives population differentiation in the skeletal genes in modern humans. Hum Mol Genet. 2010;19: 2341–2346.](http://paperpile.com/b/AKVIGO/9e36O)

109. [Chen Z. Physiological, transcriptomic and genomic mechanisms of thermal adaptation in Oncorhynchus mykiss. University of British Columbia. 2017. doi:](http://paperpile.com/b/AKVIGO/tSPIP)[10.14288/1.0340726](http://dx.doi.org/10.14288/1.0340726)

110. [do Prado FD, Vera M, Hermida M, Bouza C, Pardo BG, Vilas R, et al. Parallel evolution and adaptation to environmental factors in a marine flatfish: Implications for fisheries and aquaculture management of the turbot (Scophthalmus maximus). Evol Appl. 2018;11: 1322–1341.](http://paperpile.com/b/AKVIGO/PFema)

111. [Twomey AJ, Berry DP, Evans RD, Doherty ML, Graham DA, Purfield DC. Genome-wide association study of endo-parasite phenotypes using imputed whole-genome sequence data in dairy and beef cattle. Genet Sel Evol. 2019;51: 15.](http://paperpile.com/b/AKVIGO/zO8Gr)

112. [Li RW, Li C. Butyrate induces profound changes in gene expression related to multiple signal pathways in bovine kidney epithelial cells. BMC Genomics. 2006;7: 234.](http://paperpile.com/b/AKVIGO/QpuWq)

113. [Ringseis R, Zeitz JO, Weber A, Koch C, Eder K. Hepatic transcript profiling in early-lactation dairy cows fed rumen-protected niacin during the transition from late pregnancy to lactation. J Dairy Sci. 2019;102: 365–376.](http://paperpile.com/b/AKVIGO/DJea4)

114. [Williams JL, Dunner S, Valentini A, Mazza R, Amarger V, Checa ML, et al. Discovery, characterization and validation of single nucleotide polymorphisms within 206 bovine genes that may be considered as candidate genes for beef production and quality. Anim Genet. 2009;40: 486–491.](http://paperpile.com/b/AKVIGO/ZFGc9)

115. [Weldenegodguad M, Popov R, Pokharel K, Ammosov I, Ming Y, Ivanova Z, et al. Whole-Genome Sequencing of Three Native Cattle Breeds Originating From the Northernmost Cattle Farming Regions. Front Genet. 2018;9: 728.](http://paperpile.com/b/AKVIGO/1Xem6)

116. [Lei N, Mellem JE, Brockie PJ, Madsen DM, Maricq AV. NRAP-1 Is a Presynaptically Released NMDA Receptor Auxiliary Protein that Modifies Synaptic Strength. Neuron. 2017;96: 1303–1316.e6.](http://paperpile.com/b/AKVIGO/xCuPV)

117. [Wang Y, Harashima S-I, Liu Y, Usui R, Inagaki N. Sphingosine kinase 1-interacting protein is a novel regulator of glucose-stimulated insulin secretion. Sci Rep. 2017;7: 779.](http://paperpile.com/b/AKVIGO/80XBt)

118. [Cañadas-Garre M, Anderson K, Cappa R, Skelly R, Smyth LJ, McKnight AJ, et al. Genetic Susceptibility to Chronic Kidney Disease - Some More Pieces for the Heritability Puzzle. Front Genet. 2019;10: 453.](http://paperpile.com/b/AKVIGO/7FSno)

119. [Purfield DC, Bradley DG, Evans RD, Kearney FJ, Berry DP. Genome-wide association study for calving performance using high-density genotypes in dairy and beef cattle. Genet Sel Evol. 2015;47: 47.](http://paperpile.com/b/AKVIGO/bdycD)

120. [Ang CE, Ma Q, Wapinski OL, Fan S, Flynn RA, Lee QY, et al. The novel lncRNA lnc-NR2F1 is pro-neurogenic and mutated in human neurodevelopmental disorders. Elife. 2019;8. doi:](http://paperpile.com/b/AKVIGO/UWugu)[10.7554/eLife.41770](http://dx.doi.org/10.7554/eLife.41770)

121. [Lu W-C, Zhou Y-X, Qiao P, Zheng J, Wu Q, Shen Q. The protocadherin alpha cluster is required for axon extension and myelination in the developing central nervous system. Neural Regeneration Res. 2018;13: 427–433.](http://paperpile.com/b/AKVIGO/7sCyQ)

122. [Yang R, Fang S, Wang J, Zhang C, Zhang R, Liu D, et al. Genome-wide analysis of structural variants reveals genetic differences in Chinese pigs. PLoS One. 2017;12: e0186721.](http://paperpile.com/b/AKVIGO/Ss1pk)
