# Supplementary figures and images for: Powerful detection of polygenic selection and evidence of environmental adaptation in US beef cattle
Source: PLoS Genet. 2021 Jul 22;17(7):e1009652. doi: 10.1371/journal.pgen.1009652 (PMC8297814; doi:10.1371/journal.pgen.1009652)

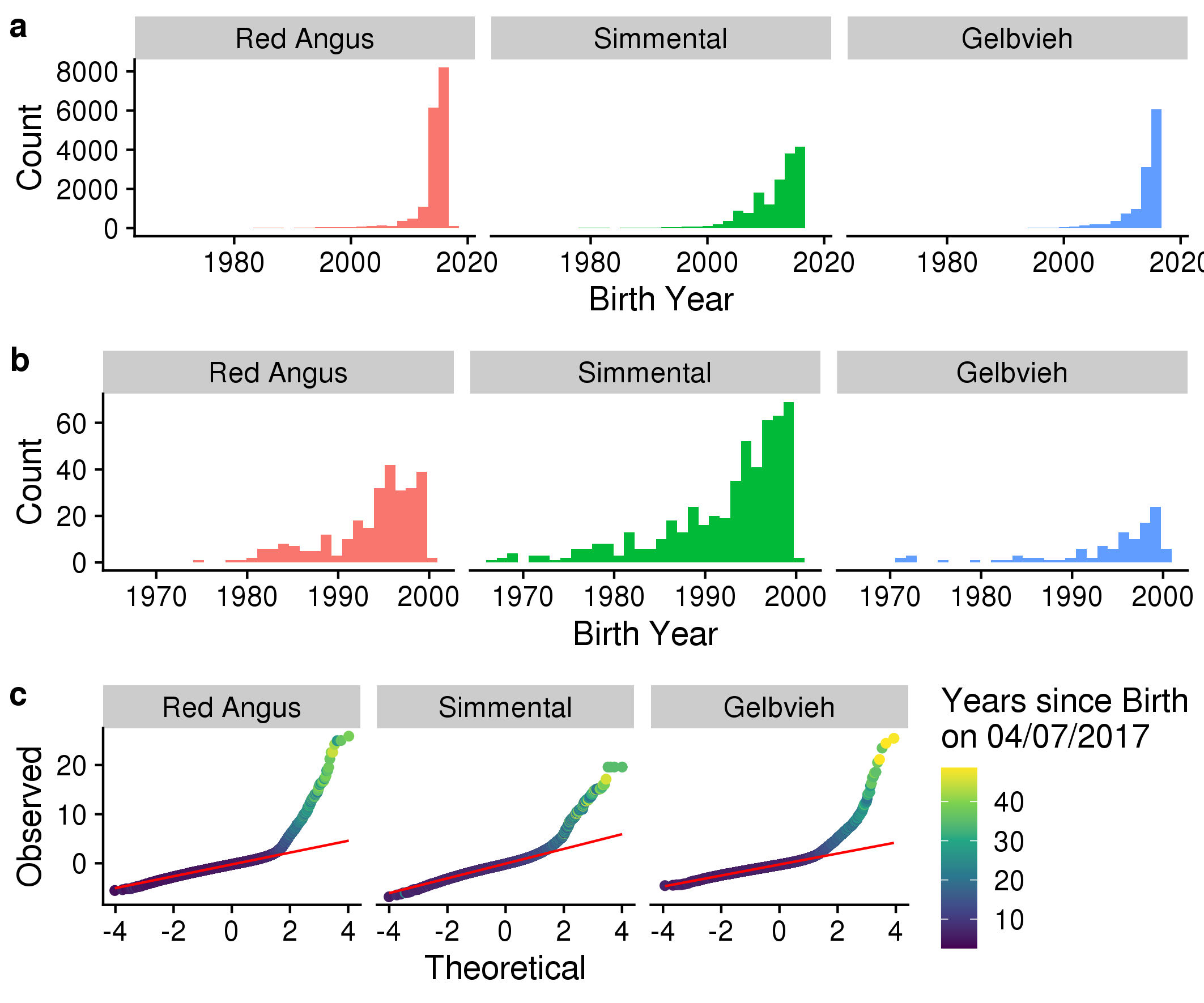

Supplement: S1 Fig — (a) Birth date histograms for complete datasets. (b) Histograms of animal birth dates born before 2000. (c) Q-Q plots of residual error from a GREML analysis of birth date in each population. Points represent individuals, and are colored by the number of years since the animal’s birth date. (TIF) [file pgen.1009652.s002.tif]

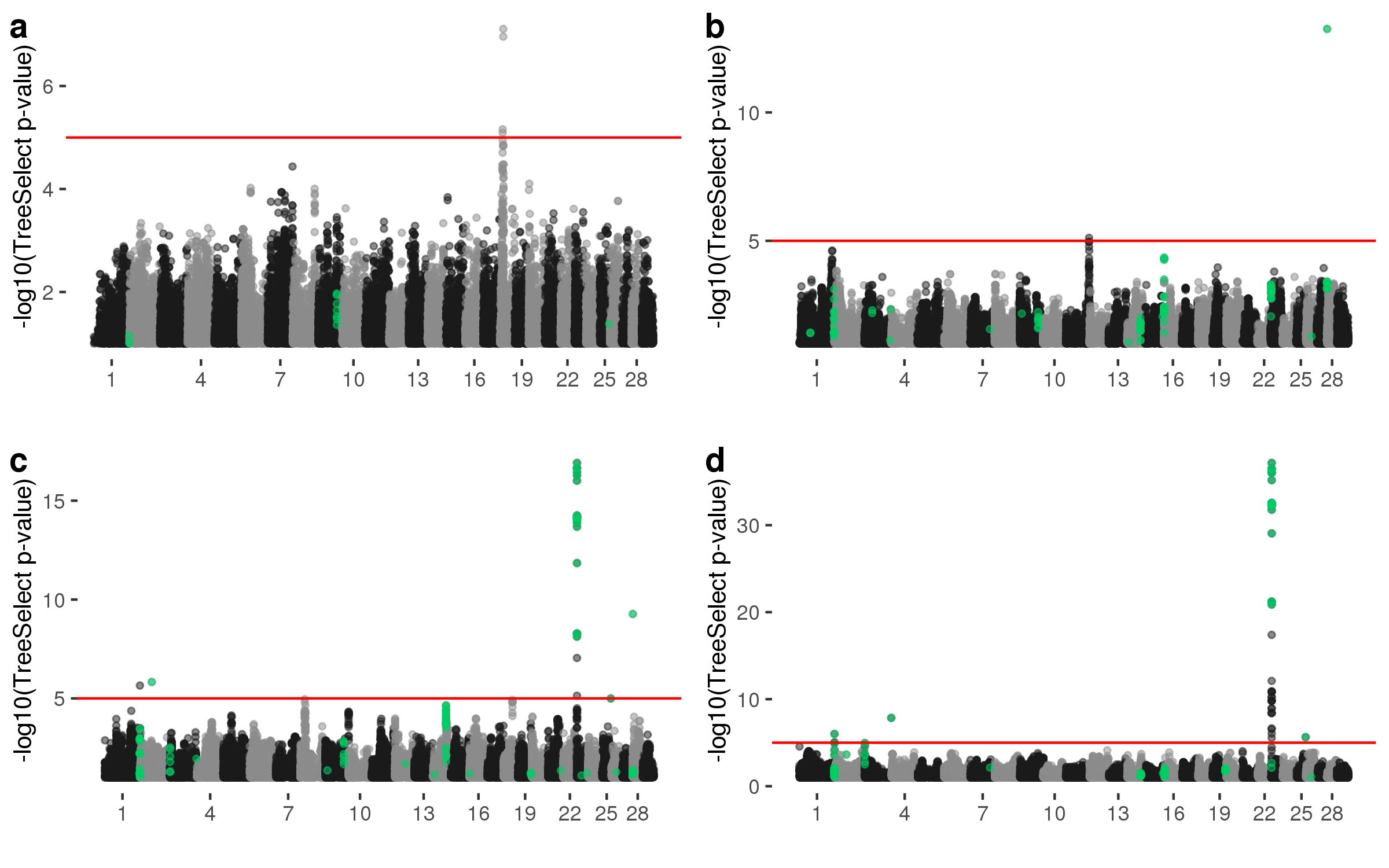

Supplement: S2 Fig — a) Single SNP -log10(p-values) for Red Angus branch of across-breed TreeSelect analysis. TreeSelect Manhattan plots for b) oldest ⅓, c) middle ⅓, and d) youngest ⅓ branches in within-breed analysis for the Red Angus population. Red line indicates significance at p < 1 x 10^−5^. Green points are SNPs that were significant (q < 0.1) in GPSM analysis of Red Angus dataset. (TIF) [file pgen.1009652.s003.tif]

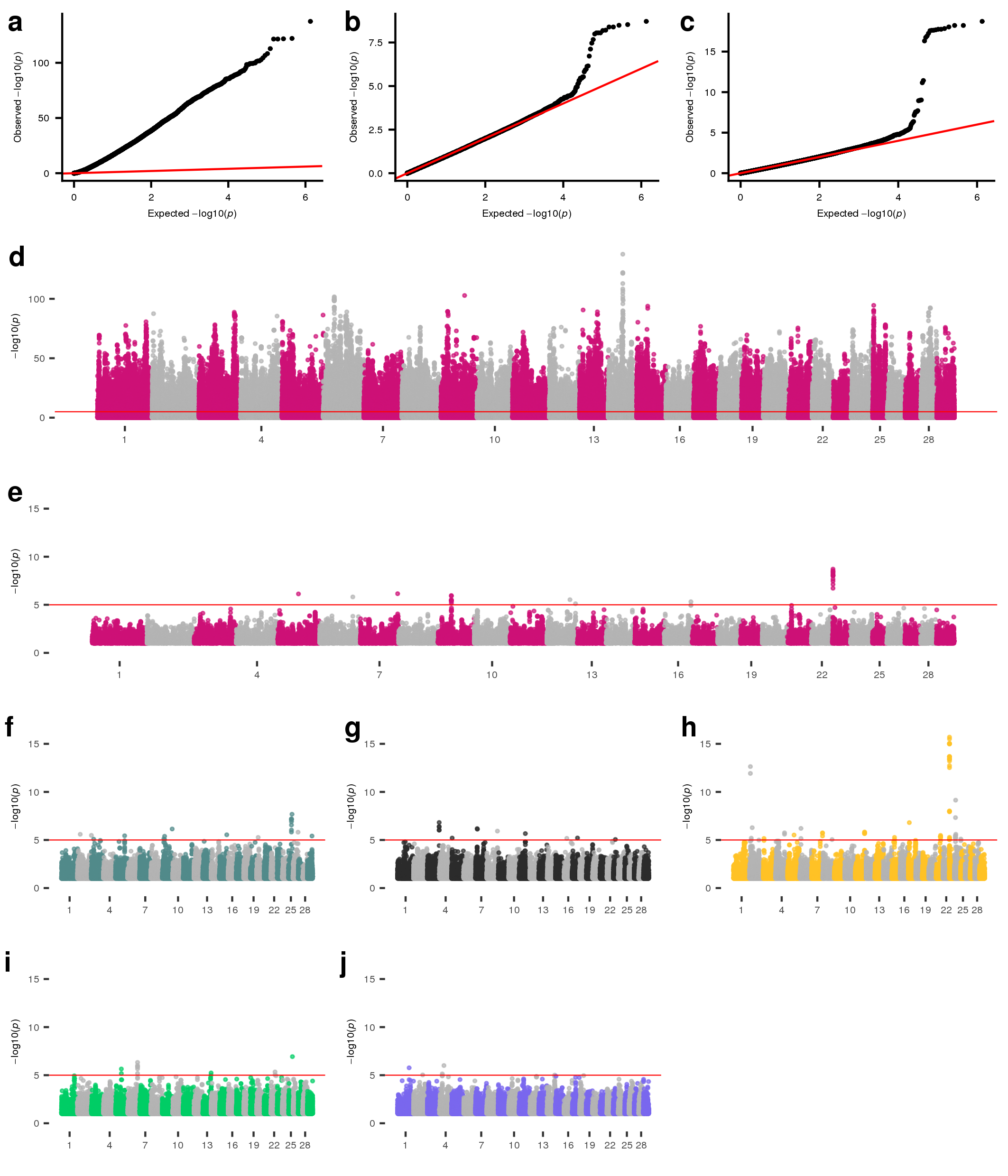

Supplement: S3 Fig — Q-Q plots for envGWAS p-values of (a) a linear model for Forested Mountains ecoregion membership, (b) a linear mixed model for Forested Mountain ecoregion membership, and (c) a multivariate linear mixed model of ecoregion membership. Univariate discrete envGWAS for (d) Forested Mountain linear model, (e) Forested Mountains linear mixed model, (f) Southeast, (g) Fescue Belt, (h) Arid Prairie, (i) High Plains, and (j) Upper Midwest & Northeast ecoregions. In all Manhattan plots the red line indicates an empirically-derived p-value significance threshold from permutation testing (p < 1×10^–5^). Note the drastically inflated p-values from the linear model in (a). Further, note that associated loci are not consistent between linear model and linear mixed model, highlighting the need to control for geographic dependency with a genomic relationship matrix. (TIF) [file pgen.1009652.s004.tif]

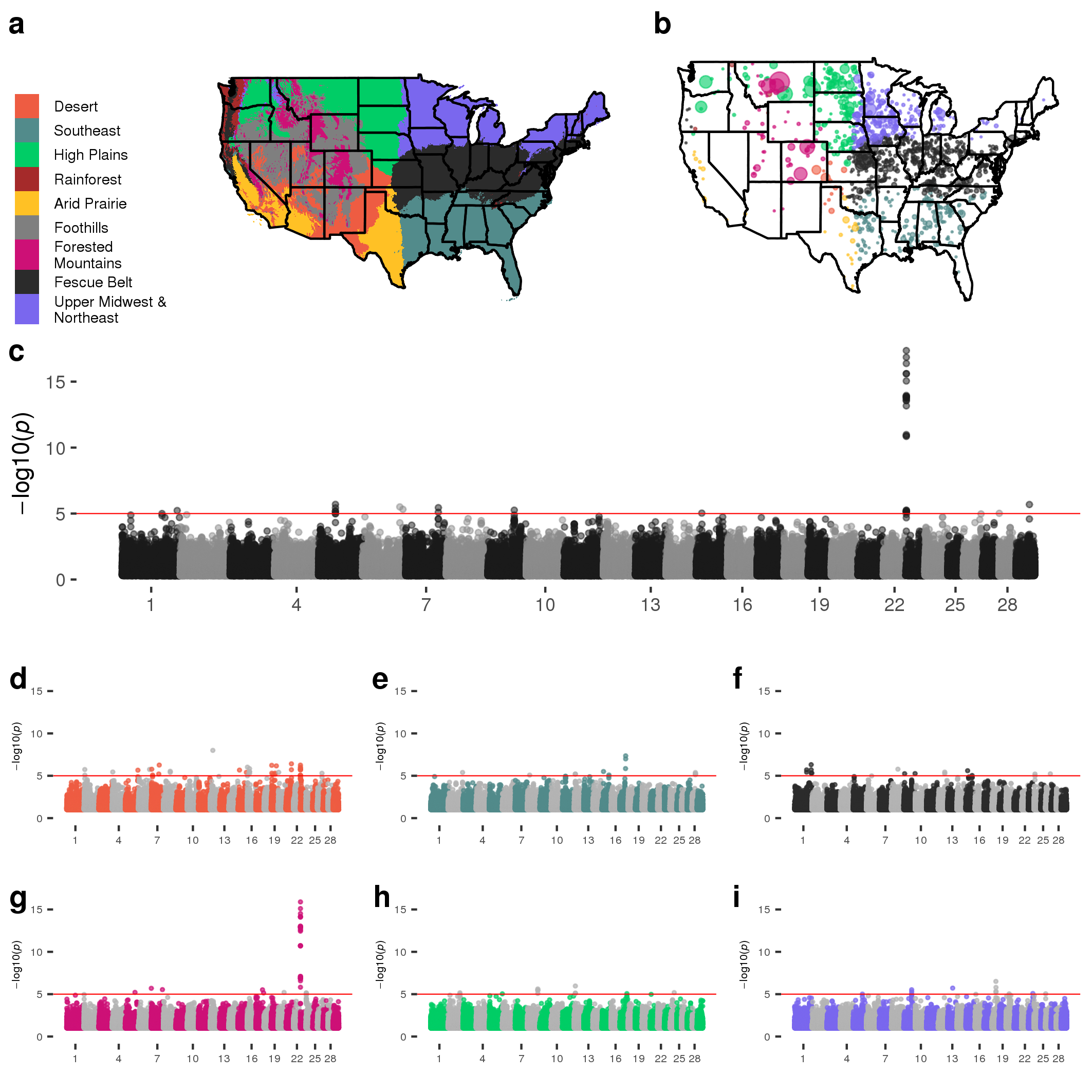

Supplement: S4 Fig — (a) Nine ecoregions of the continental United States defined by K-means clustering of 30-year normal temperature, precipitation, and elevation. (b) Locations of Simmental animals colored by breeder’s ecoregion and sized by number of animals at that location. (c) Multivariate envGWAS (case-control for regions with > 600 animals). Univariate discrete envGWAS for (d) Desert, (e) Southeast, (f) Fescue Belt, (g) Forested Mountains, (h) High Plains, and (i) Upper Midwest & Northeast ecoregions. In all Manhattan plots the red line indicates an empirically-derived p-value significance threshold from permutation testing (p < 1×10^−5^). Maps were plotted using public domain data from the US Department of Commerce, Census Bureau via the R package maps (version 3.1, https://cran.r-project.org/web/packages/maps/). (TIF) [file pgen.1009652.s005.tif]

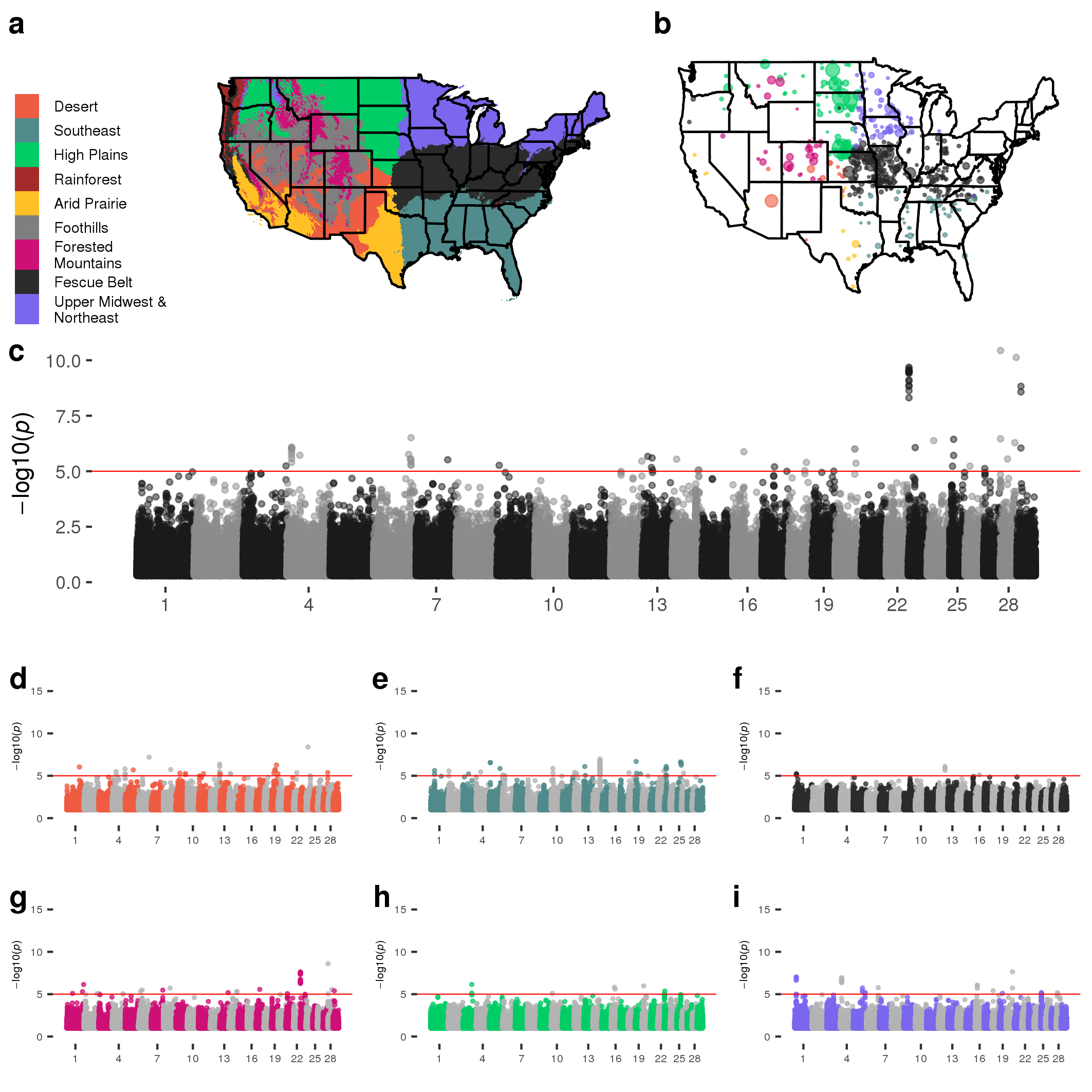

Supplement: S5 Fig — (a) Nine ecoregions of the continental United States defined by K-means clustering of 30-year normal temperature, precipitation, and elevation. (b) Locations of Gelbvieh animals colored by breeder’s ecoregion and sized by number of animals at that location. (c) Multivariate envGWAS (case-control for regions with > 600 animals). Univariate discrete envGWAS for (d) Desert, (e) Southeast, (f) Fescue Belt, (g) Forested Mountains, (h) High Plains, and (i) Upper Midwest & Northeast ecoregions. In all Manhattan plots the red line indicates an empirically-derived p-value significance threshold from permutation testing (p < 1×10^−5^). Maps were plotted using public domain data from the US Department of Commerce, Census Bureau via the R package maps (version 3.1, https://cran.r-project.org/web/packages/maps/). (TIF) [file pgen.1009652.s006.tif]

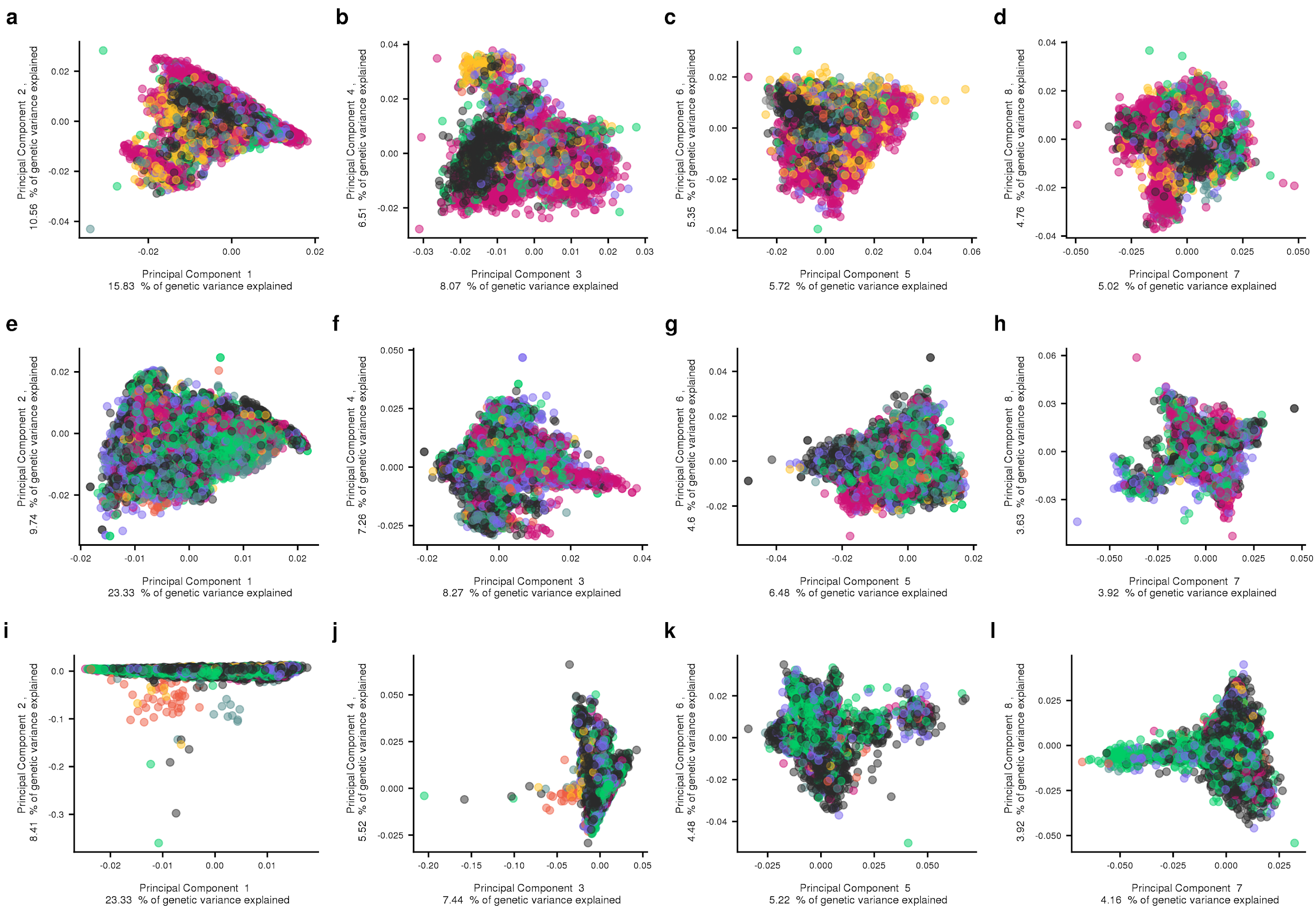

Supplement: S6 Fig — Plots for Red Angus (A-D), Simmental (E-H), and Gelbvieh (I-L). Points indicate individuals, colored by their assigned ecoregion. (TIF) [file pgen.1009652.s007.tif]

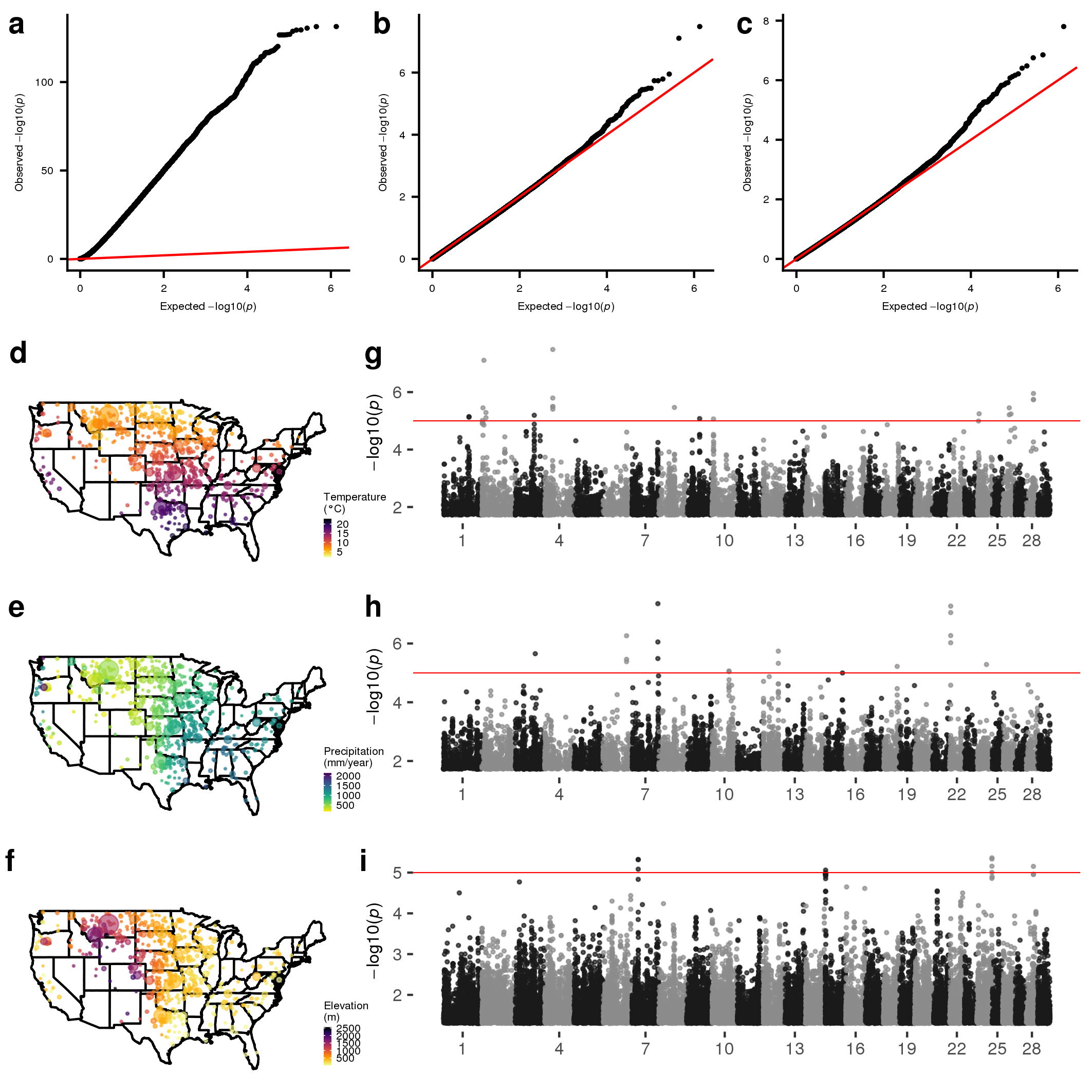

Supplement: S7 Fig — Q-Q plots for envGWAS p-values of (a) a linear model for temperature, (b) a linear mixed model for temperature, and (c) a multivariate linear mixed model of temperature, precipitation, and elevation. Geographic distributions colored by (d) temperature, (e) precipitation, (f) elevation. Manhattan plots for univariate envGWAS analysis of (g) temperature, (h) precipitation, (i) elevation. Red lines indicate permutation-derived p-value cutoff of 1×10^−5^. Maps were plotted using public domain data from the US Department of Commerce, Census Bureau via the R package maps (version 3.1, https://cran.r-project.org/web/packages/maps/). (TIF) [file pgen.1009652.s008.tif]

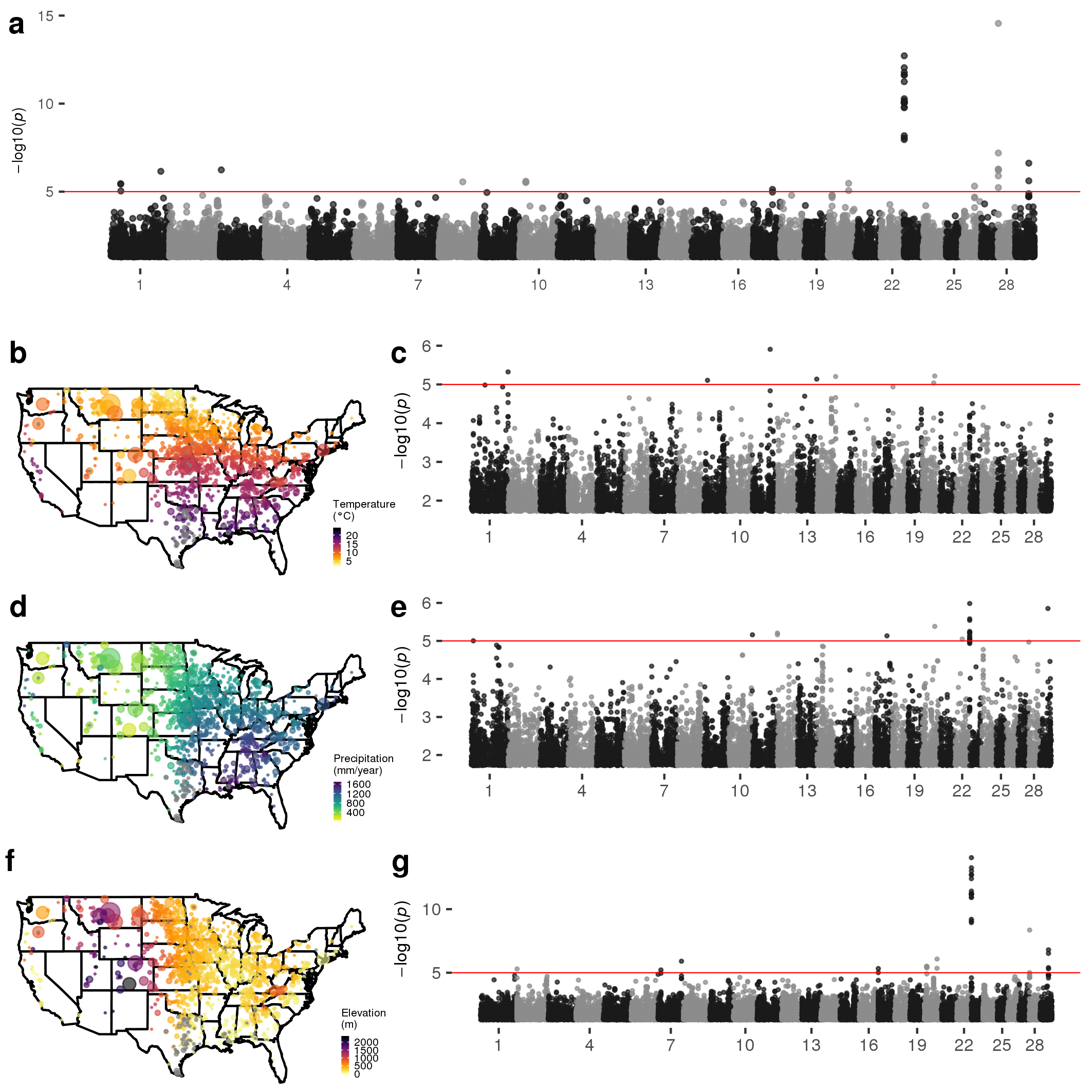

Supplement: S8 Fig — (a) Multivariate envGWAS of temperature, precipitation, and elevation for Simmental cattle. Geographic distributions colored by (b) temperature, (d) precipitation, (f) elevation. Manhattan plots for univariate envGWAS analysis of (c) temperature, (e) precipitation, (g) elevation. Red lines indicate permutation-derived p-value cutoff of 1×10^−5^. Maps were plotted using public domain data from the US Department of Commerce, Census Bureau via the R package maps (version 3.1, https://cran.r-project.org/web/packages/maps/). (TIF) [file pgen.1009652.s009.tif]

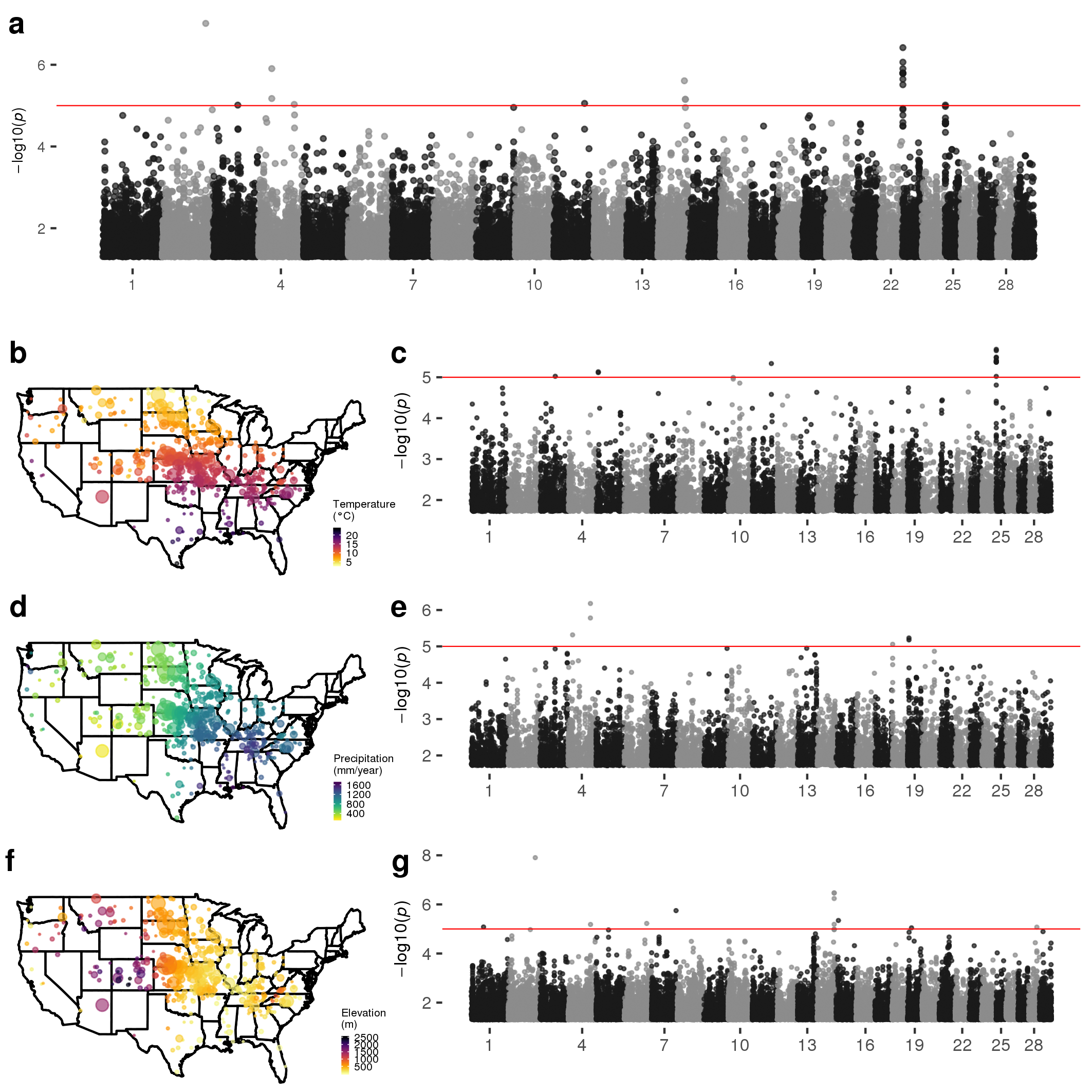

Supplement: S9 Fig — (a) Multivariate envGWAS of temperature, precipitation, and elevation for Gelbvieh cattle. Geographic distributions colored by (b) temperature, (d) precipitation, (f) elevation. Manhattan plots for univariate envGWAS analysis of (c) temperature, (e) precipitation, (g) elevation. Red lines indicate permutation-derived p-value cutoff of 1×10^−5^. Maps were plotted using public domain data from the US Department of Commerce, Census Bureau via the R package maps (version 3.1, https://cran.r-project.org/web/packages/maps/). (TIF) [file pgen.1009652.s010.tif]

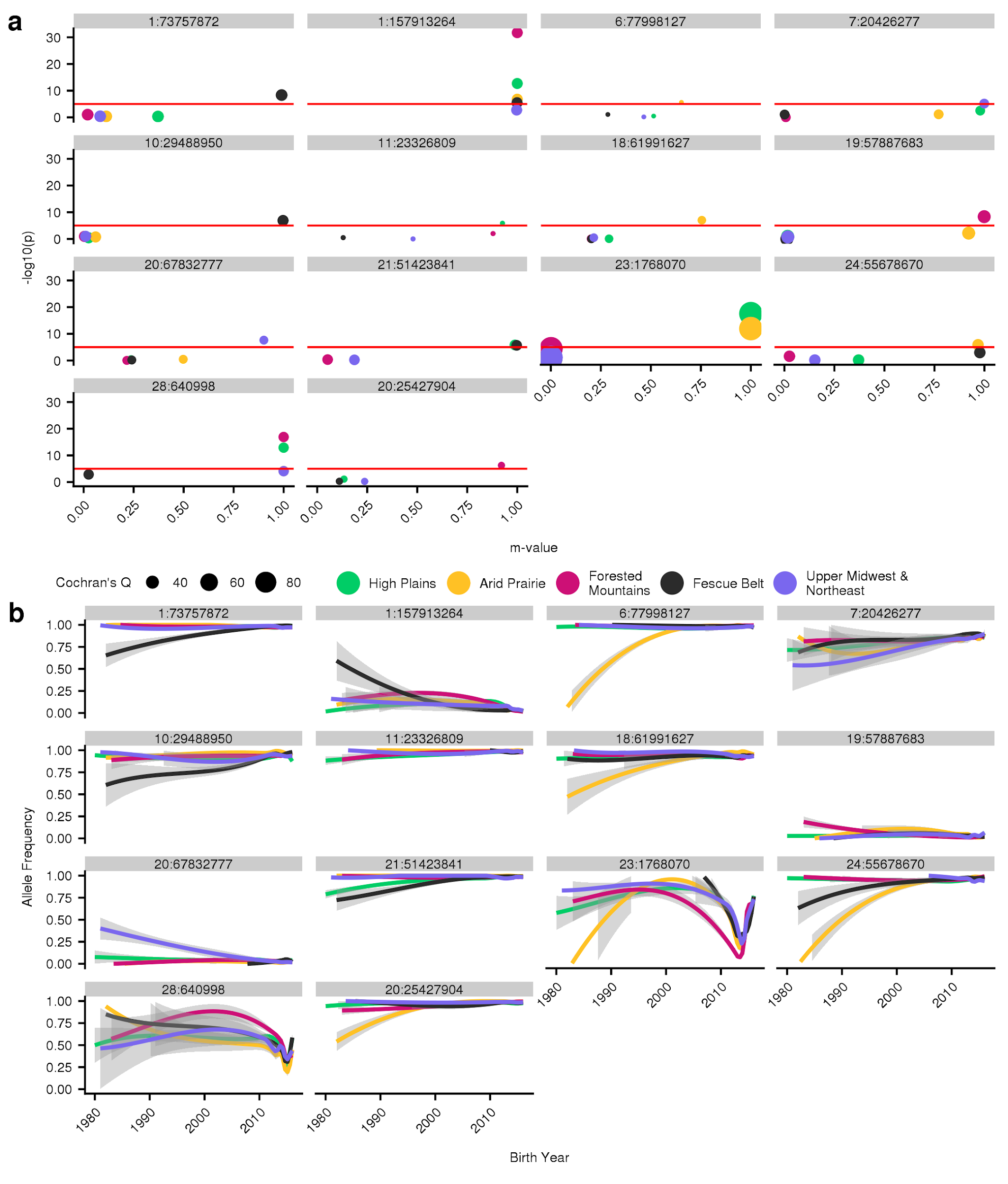

Supplement: S10 Fig — (a) PM-plots for lead SNPs of significant within-region GPSM meta-analysis (Cochran’s Q p-value > 1×10^−5^ and significant in at least one region-specific GPSM analysis p < 1×10^−5^). Each box represents the lead SNP, colored by ecoregion, and sized by Cochran’s Q value (for heterogeneity). (b) Region-specific allele frequency trajectories for lead SNPs since 1980, generated by fitting smoothed loess regression of allele frequency on birth date. Trajectories are colored by ecoregion. (TIF) [file pgen.1009652.s011.tif]

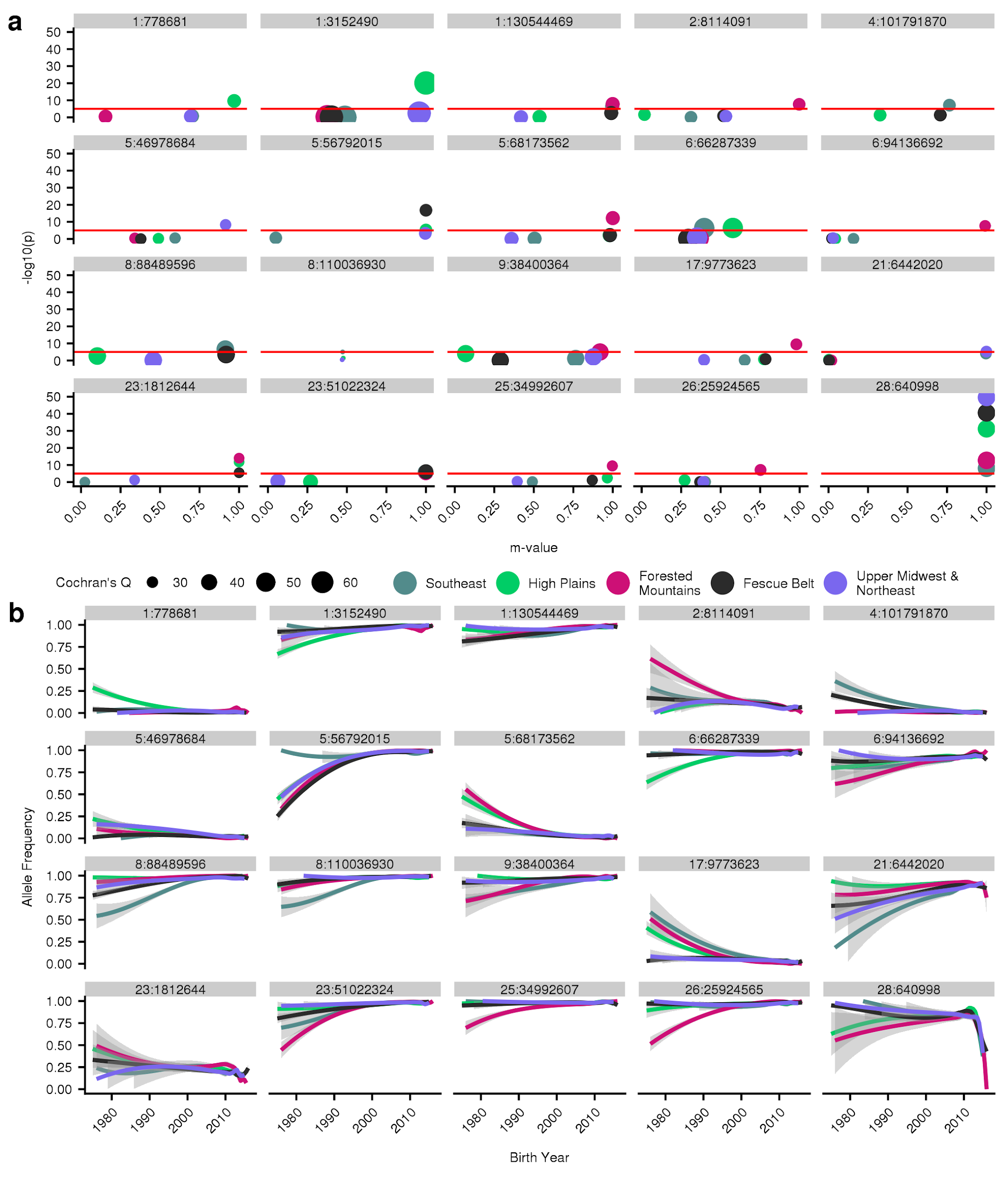

Supplement: S11 Fig — (a) PM-plots for lead SNPs of significant within-region GPSM meta-analysis (Cochran’s Q p-value > 1×10^−5^ and significant in at least one region-specific GPSM analysis p < 1×10^−5^). Each box represents the lead SNP, colored by ecoregion, and sized by Cochran’s Q value (for heterogeneity). (b) Region-specific allele frequency trajectories for lead SNPs since 1980, generated by fitting smoothed loess regression of allele frequency on birth date. Trajectories are colored by ecoregion. (TIF) [file pgen.1009652.s012.tif]

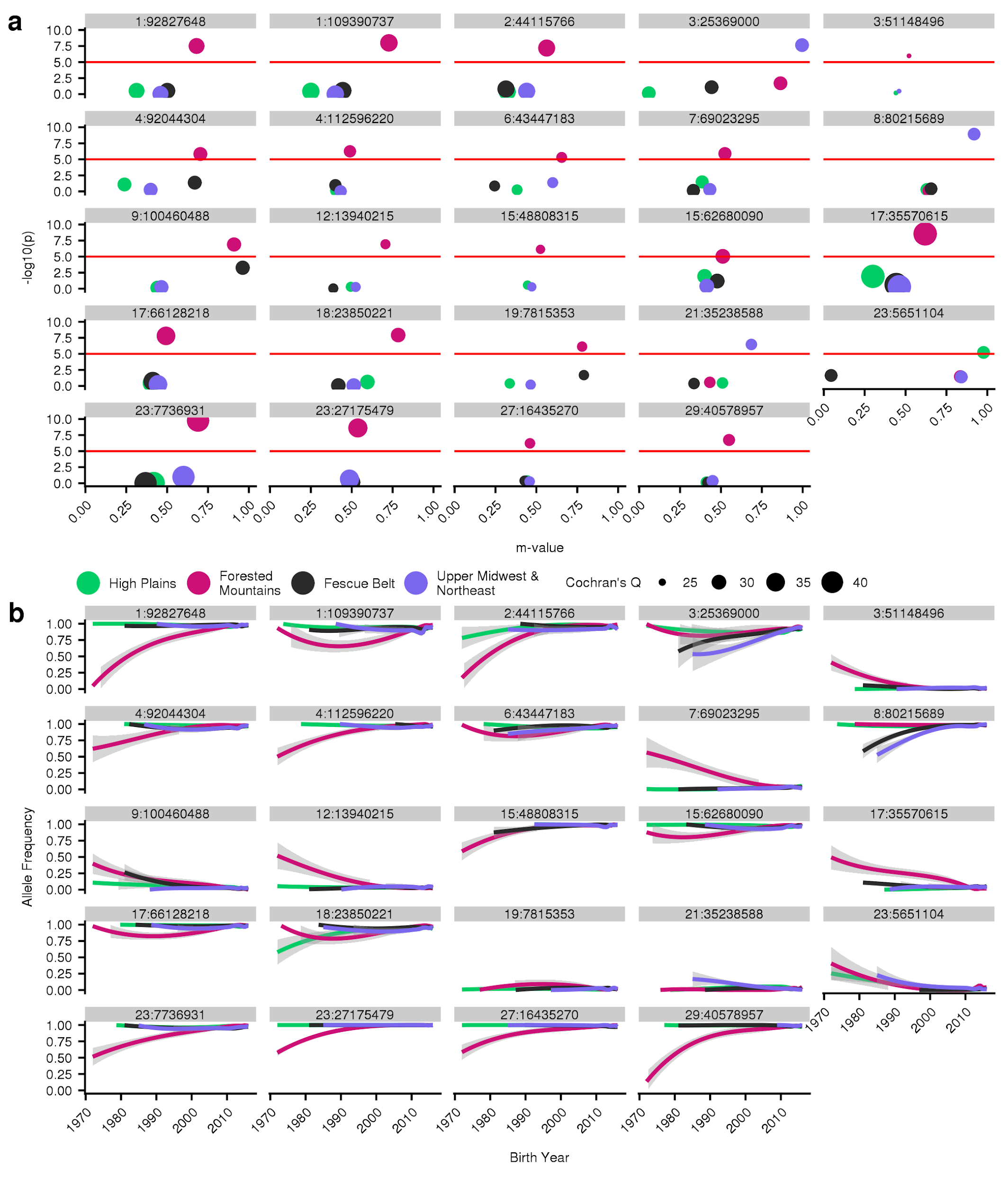

Supplement: S12 Fig — (a) PM-plots for lead SNPs of significant within-region GPSM meta-analysis (Cochran’s Q p-value > 1×10^−5^ and significant in at least one region-specific GPSM analysis p < 1×10^−5^). Each box represents the lead SNP, colored by ecoregion, and sized by Cochran’s Q value (for heterogeneity). (b) Region-specific allele frequency trajectories for lead SNPs since 1980, generated by fitting smoothed loess regression of birth of allele frequency on birth date. Trajectories are colored by ecoregion. (TIF) [file pgen.1009652.s013.tif]
